# Independent analysis of the radiation risk for leukaemia in children and adults with mortality data (1950–2003) of Japanese A-bomb survivors

**DOI:** 10.1007/s00411-012-0437-6

**Published:** 2012-11-04

**Authors:** Jan Christian Kaiser, Linda Walsh

**Affiliations:** 1 Helmholtz Zentrum München, German Research Centre for Environmental Health, Institute of Radiation Protection, 85764 Oberschleissheim, Germany; 2 Department Radiation Protection and Health, Federal Office for Radiation Protection, 85764 Oberschleissheim, Germany; 3 The Faculty of Medical and Human Sciences, University of Manchester, Manchester, UK

**Keywords:** Leukaemia mortality, Radiation risk, A-bomb survivors, Nonlinear dose response, Multi-model inference

## Abstract

A recent analysis of leukaemia mortality in Japanese A-bomb survivors has applied descriptive models, collected together from previous studies, to derive a joint excess relative risk estimate (ERR) by multi-model inference (MMI) (Walsh and Kaiser in Radiat Environ Biophys 50:21–35, 2011). The models use a linear-quadratic dose response with differing dose effect modifiers. In the present study, a set of more than 40 models has been submitted to a rigorous statistical selection procedure which fosters the parsimonious deployment of model parameters based on pairwise likelihood ratio tests. Nested models were consequently excluded from risk assessment. The set comprises models of the excess absolute risk (EAR) and two types of non-standard ERR models with sigmoidal responses or two line spline functions with a changing slope at a break point. Due to clearly higher values of the Akaike Information Criterion, none of the EAR models has been selected, but two non-standard ERR models qualified for MMI. The preferred ERR model applies a purely quadratic dose response which is slightly damped by an exponential factor at high doses and modified by a power function for attained age. Compared to the previous analysis, the present study reports similar point estimates and confidence intervals (CI) of the ERR from MMI for doses between 0.5 and 2.5 Sv. However, at lower doses, the point estimates are markedly reduced by factors between two and five, although the reduction was not statistically significant. The 2.5 % percentiles of the ERR from the preferred quadratic-exponential model did not fall below zero risk in exposure scenarios for children, adolescents and adults at very low doses down to 10 mSv. Yet, MMI produced risk estimates with a positive 2.5 % percentile only above doses of some 300 mSv. Compared to CI from a single model of choice, CI from MMI are broadened in cohort strata with low statistical power by a combination of risk extrapolations from several models. Reverting to MMI can relieve the dilemma of needing to choose between models with largely different consequences for risk assessment in public health.

**Electronic supplementary material:**

The online version of this article (doi:10.1007/s00411-012-0437-6) contains supplementary material, which is available to authorized users.

## Introduction

In a recent analysis of leukaemia mortality in the Japanese life span study (LSS) cohort of A-bomb survivors, a joint radiation risk has been derived from a group of several models by applying the technique of multi-model inference (MMI) (Walsh and Kaiser 2011). Reduction of bias from relying on a single model for risk assessment constitutes the main virtue of MMI. Application of MMI can produce more reliable point estimates and improves the characterisation of uncertainties (Burnham and Anderson 2002). Walsh and Kaiser (2011) have chosen models for a so-called group of Occam, after a review of the relevant literature in radio-epidemiology. The group contained those models which were deemed adequate for joint risk inference (Hoeting et al. 1999; Kaiser et al. 2012). They were then ranked according to the Akaike Information Criterion (AIC) which penalises models with many parameters. A joint risk estimate is given by the mean of model-specific estimates with AIC-based weights, and confidence intervals (CI) are calculated by approximate methods.

For the models discussed in Walsh and Kaiser (2011), parameter parsimony was not always the main concern of model authors so that highly parametrised models had received negligible weights in the weighting process. This intrinsic feature of MMI was criticised by Richardson and Cole (2012). They argued that models with explanatory variables which *may* have an impact on the radiation risk are not considered adequately. In their reply, Walsh et al. (2012) cautioned against the use of model parameters which are not sufficiently supported by the data. Based on the hypothetical problem posed by Richardson and Cole (2012), Walsh et al. (2012) illustrated that models, which contain parameters with weak statistical support, may cause misleading point estimates of the risk. In other examples, over-parametrised models may have little impact on point estimates but can still inflate uncertainty ranges artificially. This side-effect contorts risk assessment in radiation protection if an accurate determination of uncertainties is desired. Such desire is brought forward in court cases related to compensation claims for detrimental health effects from occupational radiation exposure (Niu et al. 2010). For example, decisions in USA courts are sometimes based on the 99 % CI of the probability of causation for cancer in a specific organ (Kocher et al. 2008).

Thus, the criterion for the choice of models for MMI in the study of Walsh and Kaiser (2011) has been changed here, so that the advice of Walsh et al. (2012) is taken seriously. Instead of picking models from peer-reviewed literature without further qualifications, potential candidate models are now submitted to a rigorous statistical selection protocol. Such a protocol has been introduced by Kaiser et al. (2012) and applied to the selection of both descriptive and mechanistic breast cancer models for joint risk inference.

All models considered in Walsh and Kaiser (2011) include a linear-quadratic dose response with different combinations of explanatory variables such as sex, age at exposure and attained age to modify the dose response of the risk. A linear-quadratic response is also preferred in the LSS studies on leukaemia incidence (Preston et al. 1994) and mortality (Preston et al. 2004). It is recommended by committees BEIR VII (NRC 2006), ICRP (Valentin 2007) and UNSCEAR (2008) after consideration of a sizeable number of leukaemia risk studies.

Although the linear-quadratic response can be regarded as the accepted standard in the radio-epidemiology of leukaemia, a number of non-standard responses have been tested motivated by earlier investigations. Little et al. (1999) found that a quadratic-exponential response yielded optimal fits when applied to LSS leukaemia incidence data. Preston et al. (1994) applied a model of two linear dose responses, represented by two line spline functions with a changing slope at a break point, as an alternative to the linear-quadratic response. Explicitly, nonlinear dose responses with sigmoidal forms have also been investigated. They are well-known in toxicology (Hodgson 2010) and are applied in radiation biology to describe normal tissue damage, i.e., of the skin (Hall 2006).

It is emphasised here that the choice of candidate models is on no account exhaustive and that a possible inclusion of non-standard models into Occam’s group is mainly justified by goodness-of-fit criteria.

The assignment of weights to risk models is also practised to transport organ-specific risk estimates from the LSS cohort to western populations, if no information on the radiation risk in Caucasian cohorts is available. However, committees BEIR VII (NRC 2006) and ICRP (Valentin 2007) support different approaches to combine an excess absolute risk (EAR) model and an excess relative risk (ERR) model with weights quantified by expert judgement. In any case, adequate transfer models must provide a good description of the risk in the population of origin. The relevance of this statistical criterion for risk transfer concerning leukaemia will be highlighted by the present study.

Past studies of the leukaemia risk at low doses for young attained ages and ages at exposure were performed for settlements in the vicinity of nuclear power stations (NPP) (Laurier et al. 2008; Kaatsch et al. 2008) and to estimate the proportion of cases induced by computer tomography (CT) scans (Pearce et al. 2012) or natural background radiation (Wakeford et al. 2009; Little et al. 2009; Kendall et al. 2012). Investigations in these fields and, additionally, ongoing risk assessment for residents near the Japanese Fukushima Daiichi NPP may benefit from both risk estimates with stronger support of the data and a more comprehensive quantification of uncertainties, which are the aim of the present study.

## Materials and methods

### Epidemiological data set

The present study is closely related to the study of Walsh and Kaiser (2011) which used LSS mortality data from 1950 to 2000. After it appeared, the LSS data have been updated with an extended follow-up to 2003 in Report 14 (Ozasa et al. 2012). To provide an analysis with the most recent data set, all results reported by the present study are based on LSS Report 14. The updated data set comprises 86,611 subjects, 318 leukaemia deaths (including 22 cases in 2001–2003) and 3,294,282 person years (including 109,927 person years in 2001–2003). The person-year weighted means are 22 year for age at exposure, 50 year for attained age, 58 year for age of cases and 134 mSv for the weighted dose to bone marrow with a factor of ten for the relative biological effectiveness (RBE) of neutrons. The RBE value depends on the radiation field and the detrimental health effect under observation. For leukaemia, an estimation is difficult and produces very large CI (Little 1997; Hunter and Charles 2002). The LSS cohort data in file lss14.csv are available for download from the website of the Radiation Effects Research Foundation (RERF) in Japan (http://www.rerf.or.jp).

### The MECAN software package

The analysis has been performed with the MECAN software package which is available from the corresponding author by request (Kaiser 2010). A user manual, regression control files and an executable to repeat the present analysis are included. MECAN is executed in a terminal on a command line under Linux or Windows. To implement risk models other than those applied here, a minimal knowledge of the C++ programming language is required. The code includes the C++ library MINUIT2 (Moneta and James 2010) from CERN which minimises the Poisson likelihood. Pre-processing of the grouped data, regression, comparison of observed and expected cases, and simulation of uncertainty intervals can all be performed in one run. The calculation of risk estimates from MMI is automated with shell script files which contain the set of required commands.

Results from MECAN for the preferred models of the present study and of the study by Walsh and Kaiser (2011) have been cross-checked by independent calculations with the EPICURE package (Preston et al. 1993). Deviances from the two packages differed by around 10^−3^ points. Relative differences of estimates for model parameters, Wald-type standard errors and CI from the likelihood profile fell below 10^−2^. Relative differences in the entries of the parameter correlation matrices exceeded one per cent in some cases.

### Baseline model

The model for the baseline mortality rates

1 $$ \begin{aligned} h_0(s,c,a,e) &= \exp \{b_0 + b_s s + b_c c \\ &\quad+ b_{a_1} \ln\left(a/55\right) + b_{a_2} \ln^2\left( a/55\right) \\ &\quad+ b_{e_1} \left( e - 30\right) + b_{e_2} \left( e - 30\right)^2 \} \end{aligned} $$

applies the same functional form as the models of the UNSCEAR committee and of Little et al. (2008) (see Table 8 of Walsh and Kaiser 2011). The parameter *b*_0_ represents a constant factor, parameters *b*_*s*_ and *b*_*c*_ account for rate differences by sex (males *s* =  −1 and females *s* =  +1) and city, i.e. Hiroshima (*c* =  −1) and Nagasaki (*c* =  +1). Parameters $b_{a_1}$ and $b_{a_2}$ quantify variations of the rates with attained age. Parameters $b_{e_1}$ and $b_{e_2}$ depend on age at exposure which for the acute exposure of the A-bomb survivors serves as a surrogate for dependence on birth cohort to account for secular trends in baseline rates. The present baseline model consumes seven adjustable parameters.

### Model selection protocol

The selection protocol of Kaiser et al. (2012) has been applied here. It starts with step-by-step attempts to optimise the baseline model in Eq. (1) with exposure-related features contained in a set of candidate models. Parameters are added individually or in groups and retained, if the nested model with the additional parameter(s) survived a likelihood ratio test (LRT) against the model of origin. For nested models, the difference between their deviances is χ^2^-distributed (Claeskens and Hjort 2008; Walsh 2007). The number of degrees of freedom for the difference is equal to the difference in the number of parameters. A model with one additional parameter is considered an improvement over the model without this parameter with a 95 % probability if the deviance is lowered by at least 3.84 points. The probability threshold is set relatively high to avoid inclusion of spurious features in risk models (Anderson et al. 2001; Walsh et al. 2012).

**Fig. 1:**
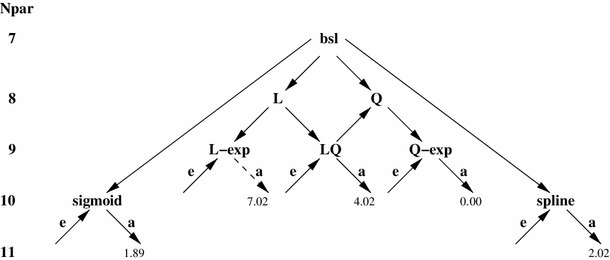
Flow chart of model selection. Models are grouped in rows pertaining to equal number of model parameters *N*_*par*_. The protocol starts with the baseline model bsl (*top*), arrows point to models which survived a pairwise LRT on the 95 % level. Dose effect modifiers are annotated as *e* for age at exposure and as *a* for attained age. AIC differences to the preferred model Q-exp with dose effect modifier for *a* are given for all models surviving the last round of tests. Model L-exp with dose effect modifier for *a* is discarded because its $\Updelta AIC$ exceeded 5.99 (*dashed arrow line*)

In the first round, various versions of the dose response are tested which are shown schematically in the flow chart of Fig. 1. To retain clarity, not all tested models are shown. A second round would involve improvements with dose effect modifications by explanatory variables such as sex, age at exposure or attained age, an example is given in Eq. (4). After passing an LRT, a model is kept for further rounds of testing. It may join Occam’s group, if improvements are no longer possible. Defeated models are rejected for risk assessment. In Fig. 1, a defeated model is identified by at least one arrow pointing away from it. Models surviving the last round of tests ‘see’ only arrowheads. More details of the protocol are given in Kaiser et al. (2012).

In the present analysis, an additional selection criterion prevents the overpopulation of Occam’s group with models of negligible influence. Based on the Akaike Information (Akaike 1973; Burnham and Anderson 2002)

2 $$ \hbox{AIC} = dev + 2\;N_{par}, $$

where *dev* denotes the Poisson deviance and *N*_*par*_ denotes the number of parameters, a weight $1/[1+\exp(-\Updelta {\rm AIC}_k/2)]$ can be constructed for the pairwise comparison of the preferred model with AIC_0_ and model *k* with AIC_*k*_, where $\Updelta {\rm AIC}_k = {\rm AIC}_k -{\rm AIC}_0.$ If this weight fell below 5 % (or $\Updelta {\rm AIC}_k$ exceeds 5.99), the corresponding model *k* was not used for risk assessment (Hoeting et al. 1999; Walsh 2007). Note, that the second criterion does not constitute a statistical test (Burnham and Anderson 2002, p 84). After its application, the L-exp model with dose effect modifier for attained age has been discarded (see Fig. 1).

At the end of the selection procedure, Occam’s group of non-nested risk models with enough relevance for risk assessment has been established for use in the MMI_*SP*_ analysis. 1

### Candidate models for Occam’s group

From the outset, the dose response of candidate models is constrained to yield a zero excess risk at zero dose and to rise monotonously with an increasing dose. Models with hormetic dose responses have not been tested but would have been admitted into Occam’s group if they qualified. Apart from these preconditions, admission to Occam’s group is achieved solely by sufficient goodness-of-fit.

Improvements of the baseline model from Eq. (1) have been attempted with three types of dose responses ‘LQ-exp’, ‘sigmoid’ and ‘spline’ (see Fig. 1) for both EAR and ERR models. The complete dose response of the LQ-exp model took the form (α *d* + β *d* ^2^)exp(−γ *d*). To account for random errors, the dose-squared covariable has been multiplied with a factor of 1.12 (Walsh and Kaiser 2011; Pierce et al. 1990). Sub-models with all seven possible combinations of the dose–response parameters α, β and γ have been tested but only the two parameter combinations α, β (sub-model LQ for linear-quadratic) and β, γ (sub-model Q-exp for quadratic-exponential) survived the series of LRTs. Cubic-exponential or quadratic-exponential models did not yield better fits than the Q-exp model. But a model with a sigmoidal response (which progresses from small beginnings and levels off at high doses) and a model with two linear dose responses, connected by a break point at dose *d*_*k*_ (termed spline model), could also be added to Occam’s group.

To perform valid LRTs, two continuous derivatives (i.e. a C2 condition) of the Poisson deviance with respect to the model parameters are required (Schervish 1997). All but one model apply parametric functions which are twice continuously differentiable. For the spline model, it is not obvious that the C2 condition is fullfilled for derivatives with respect to the break point *d*_*k*_. Therefore, the region around the minimum of the Poisson likelihood as a function of *d*_*k*_ has been scanned numerically by fixing *d*_*k*_ at different values and re-fitting the remaining parameters. The scan revealed a slightly tilted paraboloid so that both derivatives are indeed continuous. The minimum is reached at *d*_*k*_ = 0.36Sv (σ CI_*LP*_ 0.28; 0.52). The CI_*LP*_ are calculated from the likelihood profile with the MINOS routine of MINUIT2. A graphical evaluation of the numerical scan yielded the same values.

Dose effect modifiers of sex *s*, age at exposure *e* and attained age *a* have been tested separately and in combination but only the modifier $\exp\left(\varepsilon \ln\frac{a}{55}\right)$ has been accepted in all three types of dose responses shown in Fig. 1. The difference between males and females was not significant for all selected ERR models in contrast to the results of the (discarded) EAR models.

### Determination of model-specific risk estimates and confidence intervals

A best risk estimate for a single model is calculated with the set of parameter estimates which minimises the likelihood. To determine the corresponding CI, a probability density function (pdf) with 10,000 entries is generated by Monte-Carlo simulation which accounts for uncertainty ranges and pairwise correlations of all adjusted parameters. Two percentiles, corresponding to the required level of confidence (i.e. 95 %), are adopted as upper and lower CI.

To meet the requirement of a symmetric parameter correlation matrix as the backbone of the Monte-Carlo simulation, each parameter-specific pdf must ideally follow a Gaussian distribution. As a necessary precondition, the σ CI_*LP*_, calculated from the likelihood profile, should lie symmetrically around the best parameter estimate. The precondition is fullfilled for the baseline model given in Eq. (1) which is used by the models of Occam’s group. All parameters of the ERR in the LQ and the Q-exp model show symmetric CI_*LP*_ to a good approximation if models are centred at *e* = 30 and *a* = 55 (see Table 1). However, models centred at young ages at exposure and attained ages exhibit markedly skewed CI_*LP*_ for the linear and the quadratic term in the *ERR*(*d*). The ERR parameters *a* and *b* of the sigmoid model possess asymmetric CI_*LP*_ with ratios of 0.7 and 2 between lower and upper bound but the asymmetry did not disappear for centring at different values. The spline model had symmetric CI_*LP*_ for the two linear risk coefficients but the break point *d*_*k*_ showed asymmetric CI_*LP*_ for all tested combinations of centring. To calculate CI with Monte-Carlo simulation for all five combinations of *e* and *a*, the models have been centred at *e* = 30 and *a* = 55. Although the precondition of symmetric parameter CI_*LP*_ is not fully met for two ERR parameters of the sigmoid model and one parameter of the spline model, one expects that Monte–Carlo simulations of uncertainties for these two models yield results with a moderate bias.

**Table 1:** Best parameter estimates, symmetric Wald-type $\Updelta \hbox{CI}$ from a parabolic approximation around the minimum of the likelihood function, and $\Updelta \hbox{CI}_{LP}$ from the actual likelihood profile for the preferred Q-exp model; to facilitate the assessment of symmetry, $\Updelta \hbox{CI}$are given as distances from the best parameter estimate in the standard σ range

Name	Unit	Best estim.	Wald-type $\Updelta \hbox{CI}$	$\Updelta\hbox{CI}_{LP}$
*b*_0_	–	−9.50	0.10	−0.11; 0.10
*b*_*s*_	–	−0.322	0.057	−0.057; 0.057
*b*_*c*_	–	−0.140	0.065	−0.066; 0.064
$b_{a_1}$	–	2.11	0.27	−0.27; 0.27
$b_{a_2}$	–	1.08	0.21	−0.21; 0.20
$b_{e_1}$	10^−3^ year^−1^	6.4	−0.46	−0.46; 0.46
$b_{e_2}$	10^−4^ year^−2^	−7.2	2.3	−2.3; 2.2
β	Sv^−2^	4.3	1.2	−1.1; 1.4
γ	Sv^−1^	−0.38	0.13	−0.13; 0.13
$\epsilon$	–	−1.62	0.35	−0.36; 0.34

Centring does not change the quality of fit, i.e., the value of the Poisson deviance and the best risk estimates. Walsh and Kaiser (2011) exploited this fact and centred the risk models at seven pairs of *a* and *e* for a more convenient calculation of uncertainties. Especially at young ages, their approach (implemented in their Method 1) yielded symmetric CI in the Monte-Carlo simulations even if the correct CI_*LP*_ from the profile likelihood were highly asymmetric. To partly make up for this bias, the simulation of CI in their MMI_*PM*_ analysis has been repeated with their models centred at *e* = 30 and *a* = 55 with approx. symmetric CI_*LP*_. Moreover, the complete parameter correlation matrix was used now to simulate parameter uncertainties instead of the fraction that pertained to the ERR part of the model. In the repeated analysis, only the four models with the highest weights (see Table 3 of Walsh and Kaiser (2011)) were applied to the data set of LSS report 14 (Ozasa et al. 2012). Now the 2.5 % percentiles of the ERR for the UNSCEAR model do not drop below zero in contrast to the results reported in Table 4 of Walsh and Kaiser (2011).

### Multi-model inference

The surviving models are ranked according to their AIC, defined in Eq. (2), and to each model *k* an AIC-related weight

3 $$ p_k = \frac{\exp(-\frac{1}{2}\Updelta {\rm AIC}_k)}{\sum_{j=0}^{M-1}\exp(-\frac{1}{2}\Updelta {\rm AIC}_j)} $$

has been assigned.

The central risk estimate from MMI is given by the AIC-weighted mean of best estimates from the models in Occam’s group. The CI of the MMI mean are derived from a joint pdf with 10,000 entries which is obtained by merging the model-specific pdf with sizes corresponding to the AIC-weight (i.e. 5,301, 2,062, 1,927 and 710 realisations from models Q-exp, sigmoid, spline and LQ, see Table 2). From the joint pdf, an approximation of the unconditional sampling variance [see Burnham and Anderson 2002, Eq. (4.3)] can be obtained. Implicitly, this pdf also accounts for model correlations.

**Table 2:** Parametric dose dependence for the ERR models of Occam’s group used in MMI, the AIC-weight is calculated from Eq. (3)

Model name	Form of *ERR*(*d*)	*N*_*par*_	Deviance	AIC-Weight
Q-exp	$\beta d^2 \exp(\gamma d)$	10	2,670.890	0.5301
Sigmoid	$\frac{A}{B + d^C}$	11	2,670.778	0.2062
Spline	$$\left\{\begin{array}{lll} \alpha_1 d & \hbox{ for }& d < d_k \\ \alpha_2 (d-d_k) &\hbox{ for }& d \geq d_k \end{array} \right.$$	11	2,670.914	0.1927
LQ	α *d* + β *d* ^2^	10	2,674.910	0.0710

## Results

For a total of 26 ERR and 16 EAR candidate models, lists of Poisson deviances, number of parameters and AIC values are given in the online resource as a PDF excerpt of an EXCEL workbook (ESM1). The AIC of the preferred EAR model was still about 11 points away from the AIC of the preferred ERR model. Thus, no EAR model fell within Occam’s group.

For the four selected models, files with model-specific data on the quality of fit, parameter estimates and CI (from both the parabolic approximation of the likelihood minimum and from the likelihood profile), the parameter correlation matrix and tables to compare observed and expected cases are added to the online resource in PDF format. The data provided allow a repetition of the MMI_*SP*_ analysis without re-fitting the corresponding models.

Table 2 presents the four ERR models in Occam’s group. Only the dose dependence *ERR*(*d*) is shown there, the final form

4 $$ {\rm ERR}(d,a) = {\rm ERR}(d)\exp\left(\varepsilon \ln\frac{a}{55}\right) $$

additionally applies a power function for attained age *a* centred at 55 year.

Compared to the previous analysis, the baseline function of both the LQ model and LQ-exp model from Schneider and Walsh (2009) was replaced by Eq. (1) with one parameter less which increased the deviance by only about one point. Accounting for the explanatory variables of sex and age at exposure yielded no significant improvements of their models so they were discarded. With these modifications, the LQ model of Schneider and Walsh (2009) morphed into the LQ model of the present analysis, which is equivalent to the UNSCEAR model considered in Walsh and Kaiser (2011). With the same modifications, and after elimination of the linear term, the LQ-exp model of Schneider and Walsh (2009) became the preferred Q-exp model of the present analysis with parameter estimates given in Table 1. The model of Little et al. (2008) was excluded from Occam’s group because the dependence on age at exposure did not survive the LRT with the UNSCEAR model.

The UNSCEAR model (termed LQ model in the present analysis) dominated the MMI_*PM*_ risk estimate in Walsh and Kaiser (2011) with a weight of 51 % (see their Table 5), but here its contribution is reduced to only 7 %. Now the Q-exp model is preferred with a weight of 53 % with a four points lower deviance than the LQ model. Inspection of tables, which compare observed and expected cases in model-specific result files (here ESM3 and ESM2) of the online resource, suggests that the Q-exp model produced slightly better fits to the data at young ages of exposure and attained ages. For example, for Poisson strata (numbered 0, 10, 20 in the result files) with person-year weighted means of *e *≃ 5 year, *a *≃ 15 y the contribution to the Poisson deviance of the Q-exp model is about 2.5 points lower compared to the LQ model. Such exposure scenarios are of enhanced interest for radiation protection and here the Q-exp model yields lower (and better supported) risk estimates than models with a linear-quadratic dose response.

**Fig. 2:**
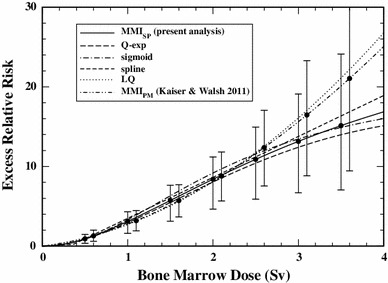
Excess relative risk (AIC-weighted mean or best estimate for separate models, with 95 % CI) for a 55-year old adult exposed at age 30 from MMI_*SP*_ (present analysis), the preferred Q-exp model, the sigmoid model, the spline model, the LQ model and from the repeated MMI_*PM*_ analysis with the four top-ranking models of Walsh and Kaiser (2011)

The quadratic term of the Q-exp model determines the response at doses <0.5 Sv, damping by the exponential term becomes important above >2.5 Sv. In the intermediate range between 0.5 and 2.5 Sv, the response is well approximated by a linear relationship (see Fig. 2). Between 2.5 and 3 Sv, nine cases have been recorded and there are only two cases above 3 Sv. The markedly different risk estimates for high doses are caused by the low statistical power in the corresponding cohort strata.

**Fig. 3:**
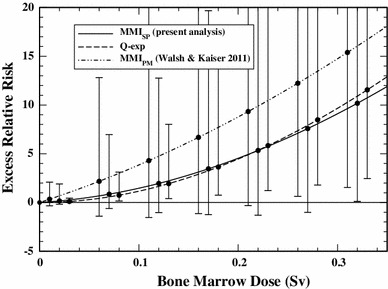
Excess relative risk (AIC-weighted mean or best estimate for model Q-exp, with 95 % CI) for a 7-year old child exposed at age 2 from MMI_*SP*_ (present analysis), the preferred Q-exp model and from the repeated MMI_*PM*_ analysis with the four top-ranking models of Walsh and Kaiser (2011)

The ERR at low doses for a 7-year-old child exposed at age 2 is shown in Fig. 3. Compared to the previous analysis, the AIC-weighted mean of the ERR from MMI_*SP*_ is reduced, i.e.,. by a factor of two at 100 mSv, although the reduction is not statistically significant. The effect of any one model is directly visible in the MMI dose response if it has a weight of more than fifty per cent. The AIC-weighted mean from MMI_*SP*_ closely follows the best estimate of the preferred Q-exp model. The additional three models cause a sizeable increase of the CI especially at low doses where a determination of the ERR implies an extrapolation to cohort strata with almost no cases (see Table 2 of Walsh and Kaiser 2011). In these regions, CI from a single model of choice underestimate the risk uncertainty by wide margins (see also Tables 3, 4).

**Table 3:** ERR (10^−2^) at 10 mSv for five combinations of age at exposure *e* and attained age *a*

Model name or MMI result	*e* = 2 *a* = 7	*e* = 2 *a* = 12	*e* = 7 *a* = 17	*e* = 12 *a* = 22	*e* = 30 *a* = 55
MMI_*PM*_	33.5	13.7	7.80	5.12	1.14
Walsh and Kaiser (2011)*	−33.5; 208	−12.4; 61.7	−7.24; 28.5	−4.78; 16.4	−1.22; 2.77
LQ	40.4	16.8	9.48	6.22	1.39
UNSCEAR (2008)	−0.753; 215	−0.370; 64.0	−0.237; 29.2	−0.167; 16.5	−0.0402; 2.80
Spline	17.7	7.34	4.15	2.72	0.609
	−36.3; 153	−12.9; 46.1	−6.98; 22.0	−4.36; 12.8	−1.00; 2.19
Sigmoid	0.851	0.358	0.204	0.135	0.0310
	1.2 × 10^−3^; 41.2	5.5 × 10^−4^; 14.9	3.2 × 10^−4^; 7.92	2.2 × 10^−4^; 5.06	5.8 × 10^−5^; 1.04
Q-exp	1.20	0.501	0.285	0.188	0.0427
(preferred model)	0.246; 5.02	0.140; 1.50	0.0953; 0.699	0.0696; 0.398	0.0184; 0.0667
$\hbox{MMI}_{SP} ^{\dagger}$	7.09 (1.41)	2.94 (0.568)	1.67 (0.317)	1.09 (0.206)	0.245 (0.0463)
(present study)	−9.02; 92.7	−3.67; 31.8	−2.01; 16.3	−1.34; 9.89	−0.323; 1.99

AIC-weighted mean for MMI or model-specific best estimate in first row, 95 % CI from Monte-Carlo simulation in second row, for MMI_*SP*_ the mean is calculated with the model-specific weights of Table 2

$^{\dagger}$ median of joint pdf from MMI_*SP*_ in brackets

$^{\ast}$ Point estimates and CI from repeated analysis (see text)

**Table 4:** ERR (10^−1^) at 100 mSv for five combinations of age at exposure *e* and attained age *a*

Model name or MMI result	*e* = 2 *a* = 7	*e* = 2 *a* = 12	*e* = 7 *a* = 17	*e* = 12 *a* = 22	*e* = 30 *a* = 55
MMI_*PM*_	38.5	15.7	8.92	5.86	1.31
Walsh and Kaiser (2011)*	−15.5; 219	−6.19; 64.7	−3.61; 29.8	−2.49; 17.0	−0.642; 2.84
LQ	43.9	18.2	10.3	6.76	1.51
UNSCEAR (2008)	2.37; 227	1.18; 66.5	0.745; 30.3	0.513; 17.2	0.125; 2.88
Spline	17.7	7.34	4.15	2.72	0.609
	−36.3; 153	−12.9; 46.1	−6.98; 22.0	−4.36; 12.8	−1.00; 2.19
Sigmoid	9.58	4.03	2.30	1.52	0.348
	0.298; 91.9	0.145; 31.4	0.0889; 15.7	0.0620; 9.56	0.0164; 1.89
Q-exp (preferred model)	11.6	4.84	2.76	1.82	0.413
(preferred model)	2.39; 48.2	1.37; 14.4	0.932; 6.67	0.683; 3.79	0.182; 0.633
MMI_*SP*_	14.6	6.10	3.47	2.28	0.515
(present study)	−8.60; 103	−3.58; 34.0	−1.97; 17.6	−1.31; 10.5	−0.312; 2.07

AIC-weighted mean for MMI or model-specific best estimate in first row, 95 % CI from Monte-Carlo simulation in second row, for *MMI*_*SP*_ the mean is calculated with the model-specific weights of Table 2

* Point estimates and CI from repeated analysis (see text)

Tables 3, 4 and 5 present the ERR from the four models of Occam’s group separately and from MMI_*SP*_ of the present analysis and of the MMI_*PM*_ analysis by Walsh and Kaiser (2011) at 10 mSv, 100 mSv and 1 Sv. At exposure of 1 Sv, both MMI analyses and all separate models yield similar estimates and CI for children, adolescents and adults.

**Table 5:** ERR at 1 Sv for five combinations of age at exposure *e* and attained age *a*

Model name or MMI result	*e* = 2 *a* = 7	*e* = 2 *a* = 12	*e* = 7 *a* = 17	*e* = 12 *a* = 22	*e* = 30 *a* = 55
MMI_*PM*_	81.8	32.9	18.7	12.3	2.76
Walsh and Kaiser (2011)*	17.0; 356	10.2; 97.7	6.24; 44.2	5.28; 24.9	1.60; 3.90
LQ	78.6	32.6	18.4	12.1	2.71
UNSCEAR (2008)	17.2; 348	10.2; 100	7.16; 44.9	5.48; 24.8	1.59; 3.81
Spline	101	42.0	23.8	15.6	3.49
	22.8; 423	13.8; 121	9.83; 54.2	7.60; 30.1	2.27; 4.69
Sigmoid	91.1	38.3	21.9	14.5	3.31
	18.7; 345	10.9; 102	7.61; 46.5	5.77; 26.3	1.56; 4.37
Q-exp (preferred model)	82.4	34.4	19.6	12.9	2.94
(preferred model)	18.2; 322	10.8; 94.4	7.45; 43.1	5.55; 24.3	1.54; 3.91
MMI_*SP*_ (present study)	87.6	36.6	20.8	13.7	3.10
(present study)	19.7; 343	11.4; 101	7.95; 46.2	5.94; 25.9	1.60; 4.31

AIC-weighted mean for MMI or model-specific best estimate in first row, 95 % CI from Monte-Carlo simulation in second row, for MMI_*SP*_ the mean is calculated with the model-specific weights of Table 2

* Point estimates and CI from repeated analysis (see text)

The situation changes at 100 mSv. Now the new preferred Q-exp model predicts a four times lower risk than the previously chosen UNSCEAR (here LQ) model. Compared to the repeated MMI_*PM*_ analysis with the four top-ranking models of Walsh and Kaiser (2011), estimates from the present MMI_*SP*_ analysis differ by a factor of 2.5 and the CI are markedly reduced.

At 10 mSv, the AIC-weighted mean of the present study no longer approximates the best estimate of the preferred Q-exp model. The mean is strongly influenced by a 30 times higher estimate of the LQ model which on the other hand acquires the lowest weight in MMI_*SP*_. To avoid this effect and to preserve the similarity between the point estimates from the preferred model and from MMI, Kaiser et al. (2012) recommend to replace the AIC-weighted mean by the median of the joint pdf, which is given in brackets in Table 3.

At doses of 10 mSv and 100 mSv, the 2.5 % percentiles from the present MMI_*SP*_ analysis include a zero risk due to the uncertainty of the spline model.

## Discussion

Little et al. (1999) analysed the dose response for three subtypes of acute myeloid leukaemia (AML), chronic myeloid leukaemia (CML) and acute lymphocytic leukaemia (ALL) separately and for all subtypes combined. Their analysis was carried out with LSS incidence data, and with two other data sets of women treated for cervical cancer (incidence) and UK patients treated for ankylosing spondilitis (mortality). From a list of 13 ERR models, using similar versions of the general LQ-exp response with dose effect modifiers, the Q-exp response has been preferred for yielding the optimal fit. They used already LRTs to discard models with statistically insignificant features. Their estimates of the coefficients β for the dose squared and γ for the exponential damping were 5.8 (95 % CI 2.7; 11) Sv^−2^ and −0.49 (95 % CI −0.76; −0.22) Sv^−1^, respectively (see their Table 5). Risk estimates for leukaemia incidence are expected to exceed those for mortality. Comparison with estimates in Table 1 shows that this relation is realised for dose $\lesssim$ 3 Sv, albeit without statistical significance.

Separate estimates for the other two data sets produced no significant risk (women with cervical cancer) or a ten times larger coefficient β (patients with ankylosing spondilitis). Comparison of risks in these different populations is complicated by the consideration that the LSS subjects were not under observation because of known diseases whereas members of the two other data sets were.

Basic tenets of MMI might be extended to address questions of risk transfer between populations which are discussed in reports of committees BEIR VII (NRC 2006) and ICRP (Valentin 2007). BEIR VII propose to transfer risks for solid cancer sites (except breast and thyroid) and for leukaemia from the LSS cohort to the US population with a linear combination of an ERR model and an EAR model. They recommend point estimates as weighted means obtained under the two models. For leukaemia and solid cancer sites (except breast, thyroid and lung), the weights of 0.7 (ERR) and 0.3 (EAR) are chosen by expert judgement based on the observation ‘that there is a somewhat greater support for relative risk than for absolute risk transport’ (see p. 276). Inconsistent with BEIR VII, ICRP recommend to apply only the EAR model of Preston et al. (1994) for leukaemia incidence.

In general, the consensus on a risk transfer model is based on a complex mix of factors, but a comprehensive consideration is beyond the scope of the present study. However, any adequate transfer model should provide a good description of the risk in the population of origin. Would goodness-of-fit criteria be allowed to assess the adequacy of a model, EAR models of leukaemia mortality would not contribute to the transfer. The best EAR model exceeds the AIC of the preferred Q-exp model by about 11 points which leads to a negligible AIC-weight. A second criterion of Bayesian information $({\rm BIC} = dev + N_{par}\ln(n_{cases}))$ is often used as an alternative to the AIC because it favours more parsimonious models (Claeskens and Hjort 2008). It is 18 points higher which constitutes strong evidence (Kass and Raftery 1995) for the rejection of the EAR model. Likewise, Little (2008) recommends to drop EAR models, but with a different rationale. Based on a comparison of risks for childhood exposure between the LSS cohort and three medically exposed groups in Europe, he observed that heterogeneity in cohort-specific EAR estimates is much higher than in ERR estimates.

A recent risk study of leukaemia (and brain tumours) after childhood exposure by CT scans reports an ERR of 36 (95 % CI 5; 120) Sv^−1^ from a purely linear model for age at exposure <22 year, dose range between 0 and 100 mSv and follow-up of 23 year (Pearce et al. 2012). The same linear model applied to the LSS incidence data (Preston et al. 1994) produced an ERR of 37 (95 % CI 14; 127) Sv^−1^ for age at exposure <20 year, dose range between 0 and 4 Sv and follow-up of 20 year (see Table 8 of the supplement to Pearce et al. (2012)).

The authors of the present study fitted a purely quadratic model to the LSS incidence data for all dose ranges which increased the deviance by 4.8 points compared to the linear model. If the overlap of dose ranges was improved by a reduction to 0–500 mSv for the LSS data, the quadratic model yielded a slightly better fit. The deviance decreased by 2.4 points compared to the linear model. Improved fits of a quadratic model at lower doses are in line with findings of the present study (mortality) and the study of Little et al. (1999) (incidence). With a coefficient of 61 (95 % CI 22; 185) Sv^−2^ for the quadratic model, the ERR at 100 mSv is six times lower than for the linear model. Using both models in MMI would still yield a reduction of the ERR by a factor of three compared to the linear model.

Nevertheless, Pearce et al. (2012) report ‘little evidence of nonlinearity of the dose response, using either linear-quadratic or linear-exponential forms of departure from linearity’, but purely quadratic responses appear not to have been tested. At this point, the present authors suggest a comparison of the CT risk with the LSS quadratic response. Should alternative dose responses, such as purely quadratic, fit the data comparably well, an even fairer comparison might account for model uncertainty in the CT cohort. In this case, reverting to MMI can relieve the dilemma of needing to choose between models with largely different consequences for issues of public health, e.g., for assessing the risk-to-benefit ratio related to a CT scan. In a wider context, MMI might be of use for statistical analysis in a number of cohort studies of CT exposure and cancer incidence which will be completed in the near future (Einstein 2012).

## Conclusions

Only models with a linear-quadratic dose response were included in the MMI analysis of Walsh and Kaiser (2011). The present analysis introduced three models with non-standard dose responses which produced significantly better fits to the data. All considered models yield very similar point estimates and uncertainties in the dose range 0.5–2.5 Sv, i.e., in cohort strata with a sufficient number of cases. Divergent predictions appear in strata with almost no cases for children and adolescents exposed to very low doses of 100 mSv and below (see Table 2 of Walsh and Kaiser 2011). Yet for purposes of radiation protection, these exposure scenarios are of increased interest. Compared to the study of Walsh and Kaiser (2011), the present MMI analysis predicts markedly lower risks with factors of two around 100 mSv and up to five for lower doses. These point estimates are considered as more reliable since they were produced with models, which describe the data slightly better notably for children and adolescents.

Besides the improvement of point estimates, a second benefit of MMI has been demonstrated. Several plausible models can be included in a more comprehensive (though not exhaustive) determination of uncertainties. Again, the benefit becomes noticeable in the above-mentioned cohort strata with low statistical power, where the risk is determined by extrapolation. Now uncertainty ranges are mainly determined by the *spread* of model-specific point estimates, whereas the model-specific uncertainty ranges are rather small. Hence, inferring uncertainties from a single model of choice may lead to a substantial underestimation. In this context, the present MMI study provides significant risks only above some three hundred mSv, whereas the 95 % CI of the preferred Q-exp model do not include a zero risk for all considered exposure scenarios.

The impact of pertinent sources of uncertainty, such as the ‘healthy survivor effect’, errors in dosimetry or misdiagnosis of cases on risk estimates has been discussed extensively in the literature, for the LSS cohort see, e.g., Little et al. (1999), Preston et al. (2003), Preston et al. (2004). Already Little et al. (1999) preferred ERR models with a Q-exp response. They did, however, not consider the additional contribution to the uncertainty which is induced by including models with other plausible dose responses into the risk analysis. In this developing field of research in radiation epidemiology, the present MMI study aims to be of help.

The model selection bias cannot be eliminated by MMI but can be markedly reduced. The bias is transferred from the level of picking a single model of choice to picking a set of candidate models. In the present analysis, this set included more than 40 models with different forms of dose responses, of which four models have been admitted to Occam’s group. Under the given rules for model selection, it appears unlikely that by broadening the basis of candidate models a considerable number of new models would enter Occam’s group. Even if new models appeared, their impact on risk estimates would be contained by the original models.

## Electronic Supplementary Material

Below is the link to the electronic supplementary material.

PDF (40 KB)

Below is the link to the electronic supplementary material.

PDF (35 KB)

Below is the link to the electronic supplementary material.

PDF (36 KB)

Below is the link to the electronic supplementary material.

PDF (36 KB)

Below is the link to the electronic supplementary material.

PDF (35 KB)

## Footnotes

1 The present study is named MMI_*SP*_ study, since models are chosen by a $\underline{S}\hbox{election}\,\underline{P}\hbox{rotocol}$. For a better distinction, the study of Walsh and Kaiser (2011) is named here MMI_*PM*_ study, since it was based on previously $\underline{P}\hbox{ublished}\,\underline{M}\hbox{odels}$.
