# Supplementary material for: Independent analysis of the radiation risk for leukaemia in children and adults with mortality data (1950–2003) of Japanese A-bomb survivors
Source: Radiat Environ Biophys. 2012 Nov 4;52(1):17–27. doi: 10.1007/s00411-012-0437-6 (PMC3579470; doi:10.1007/s00411-012-0437-6)
Supplement: Supplementary file 1 — PDF (40 KB) [file 411_2012_437_MOESM1_ESM.pdf]

Model	Model	Model	dose response param.			dose effect modifier		used		Poisson					
**no.**	**type**	**name**	**linear**	**quadr.**	**expo.**	**age at exp.**	**att. Age**	**in MMI**	**Npar**	**Deviance**	**AIC**	**ΔAIC**	**weight**	**Npdf**	**Δdev**
0	baseline	baseline	no	no	no	no	no	no	7	2898,90	2912,90	222,010			228,01
R1	LQ-exp	L	yes	no	no	no	no	no	8	2712,32	2728,32	37,434			41,43
R2	LQ-exp	Q	no	yes	no	no	no	no	8	2702,74	2718,74	27,847			31,85
R3	LQ-exp	LQ	yes	yes	no	no	no	no	9	2699,91	2717,91	27,022			29,02
R4	LQ-exp	L-exp	yes	no	yes	no	no	no	8	2702,02	2718,02	27,127			31,13
R5	LQ-exp	Q-exp	no	yes	yes	no	no	no	9	2697,58	2715,58	24,690			26,69
R6	LQ-exp	L-a	yes	no	no	no	yes	no	9	2685,72	2703,72	12,833			
R7	LQ-exp	Q-a	no	yes	no	no	yes	no	9	2679,52	2697,52	6,631			
R8	LQ-exp	LQ-a	yes	yes	no	no	yes	yes	10	2674,910	2694,910	4,020	0,0710	710	4,02
R9	LQ-exp	LQ-e	yes	yes	no	yes	no	no	10	2694,02	2714,02	23,128			23,13
R10	LQ-exp	Q-exp-a	no	yes	yes	no	yes	yes	10	2670,890	2690,890	0,000	0,5301	5301	0,00
R11	LQ-exp	Q-exp-e	no	yes	yes	yes	no	no	10	2691,32	2711,32	20,430			20,43
R12	LQ-exp	L-exp-a	yes	no	yes	no	yes	no	10	2677,91	2697,91	7,020	ΔAIC > 5.99		7,02
R13	LQ-exp	Q-exp-ae	no	yes	yes	yes	yes	no	11	2670,15	2692,15	1,260			
R14	LQ-exp	LQ-exp-a	yes	yes	yes	no	yes	no	11	2670,75	2692,75	1,860			
R15	two line spline	spline	n.a.	n.a	n.a	no	no	no	10	2698,08	2718,08	27,191			27,19
R16	two line spline	spline-a	n.a.	n.a	n.a	no	yes	yes	11	2670,914	2692,914	2,024	0,1927	1927	0,02
R17	two line spline	spline-e	n.a.	n.a	n.a	yes	no	no	11	2691,26	2713,26	22,368			20,37
R18	two line spline	spline-ae	n.a.	n.a	n.a	no	yes	no	12	2670,29	2694,29	3,399			
R19	two line spline	threshold-a	n.a.	n.a	n.a	no	yes	no	11	2671,58	2693,58	2,690			
R20	sigmoid	sigmoid	n.a.	n.a	n.a	no	no	no	10	2697,53	2717,53	26,641			26,64
R21	sigmoid	sigmoid-a	n.a.	n.a	n.a	no	yes	yes	11	2670,778	2692,778	1,888	0,2062	2062	-0,11
R22	sigmoid	sigmoid-e	n.a.	n.a	n.a	yes	no	no	11	2691,19	2713,19	22,303			20,30
R23	sigmoid	sigmoid-ae	n.a.	n.a	n.a	no	yes	no	12	2670,07	2694,07	3,183			
R24	sigmoid	tanh-a	n.a.	n.a	n.a	no	yes	no	11	2676,10	2698,10	7,210	ΔAIC > 5.99		
R25	sigmoid	Gompertz-a	n.a.	n.a	n.a	no	yes	no	12	2671,97	2695,97	5,075	Non zero excess risk at zero dose		
R26	cubic-exp	cubic-exp-a	n.a.	n.a	n.a	no	yes	no	10	2671,75	2691,75	0,860			
R27	quartic-exp	quartic-exp-a	n.a.	n.a	n.a	no	yes	no	10	2674,94	2694,94	4,050			
	**Dose effect modifier**		**Functional form**												
	a: attained age		exp(c_a ln(a/55))												
	e: age at exposure		exp(c_e (e-30))												

Model	Model	Model	dose response param.			dose effect modifier			used		Poisson		
**no.**	**type**	**name**	**linear**	**quadr.**	**expo.**	**age at exp.**	**att. Age**	**sex**	**in MMI**	**Npar**	**Deviance**	**AIC**	**ΔAIC**
A1	LQ-exp	Q-exp	no	yes	yes	no	no	no	no	9	2696,36	2714,36	23,47
A2	sigmoid	sigmoid	n.a.	n.a	n.a	no	no	no	no	10	2696,32	2716,32	25,43
A3	two line spline	spline	n.a.	n.a	n.a	no	no	no	no	10	2696,13	2716,13	25,24
A4	LQ-exp	LQ	yes	yes	no	no	no	no	no	9	2698,52	2716,52	25,63
A5	LQ-exp	Q-exp-a	no	yes	yes	no	yes	no	no	10	2689,54	2709,54	18,65
A6	sigmoid	sigmoid-a	n.a.	n.a	n.a	no	yes	no	no	11	2689,44	2711,44	20,55
A6	two line spline	spline-a	n.a.	n.a	n.a	no	yes	no	no	11	2695,86	2717,86	26,97
A8	LQ-exp	LQ-a	yes	yes	no	no	yes	no	no	10	2692,41	2712,41	21,52
										9			
A9	LQ-exp	Q-exp-ae	no	yes	yes	yes	yes	no	no	11	2682,73	2704,73	13,84
A10	sigmoid	sigmoid-ae	n.a.	n.a	n.a	yes	yes	no	no	12	2682,70	2706,70	15,81
A11	two line spline	spline-ae	n.a.	n.a	n.a	yes	yes	no	no	12	2682,58	2706,58	15,69
A12	LQ-exp	LQ-ae	yes	yes	no	yes	yes	no	no	11	2685,41	2707,41	16,52
A13	LQ-exp	Q-exp-aes	no	yes	yes	yes	yes	yes	no	12	2677,66	2701,66	10,77
A14	sigmoid	sigmoid-aes	n.a.	n.a	n.a	yes	yes	yes	no	13	2677,65	2703,65	12,76
A15	two line spline	spline-aes	n.a.	n.a	n.a	yes	yes	yes	no	13	2677,60	2703,60	12,71
A16	LQ-exp	LQ-aes	yes	yes	no	yes	yes	yes	no	12	2680,65	2704,65	13,76

Dose effect modifier	Functional form
a: attained age	exp(c_a ln(a/55))
e: age at exposure	exp(c_e (e-30))
s: sex	exp(c_s s)

	**Model**	**deviance**	**Npar**	**AIC**	**ΔAIC**	**weight**	**Npdf**
	UNSCEAR	2674,91	10	2694,91	0,00	0,5809	5809
	Little	2674,89	11	2696,89	1,98	0,2159	2159
	S+W exp	2669,61	14	2697,61	2,70	0,1505	1505
	S+W	2673,71	13	2699,71	4,80	0,0527	527
						1,0000	10000
	4 top ranking models of Walsh and Kaiser (2012) applied to LSS14 data						
