# Supplementary material for: Independent analysis of the radiation risk for leukaemia in children and adults with mortality data (1950–2003) of Japanese A-bomb survivors
Source: Radiat Environ Biophys. 2012 Nov 4;52(1):17–27. doi: 10.1007/s00411-012-0437-6 (PMC3579470; doi:10.1007/s00411-012-0437-6)
Supplement: Supplementary file 2 — PDF (35 KB) [file 411_2012_437_MOESM2_ESM.pdf]

```
-- Result protocol
   From MECAN version 0.2
   Based on MINUIT2 version 5.27.02
   In file format version rec.1.0
   Parallelisation mode 'useOpenmpDefault'
   Operation mode 'regression & analysis'
   Group data mode 'use_raw_data'

-- Control files
   MINUIT2 file 'lq.min'
   Control file 'par/leuk.par'

-- Optimisation time
   Optimisation started: Mi. Aug 15 15:04:58 2012
   Optimisation stopped: Mi. Aug 15 15:12:53 2012
           Elapsed time: 00:07:55 or 475 secs

-- Raw data summary
   Grouped data read from file 'lss14.csv'
   Created 53782 Poisson cells with 8 categories
     for organ/organ group 'leukemia'
   Counted          86611 persons
   Counted      3294282.3 person years
   Counted            318 cases
   Case counts pertain to end point(s) for 1 organ/organ groups
   End point no.  1 'Leukemia 204-208'
   Organ dose 'marrow10' with Id 9

-- Stratification summary
   Created 53782 Poisson cells with 5 categories
     for organ/organ group 'leukemia'
   Category  'city' with Id  0
   Category   'sex' with Id  1
   Category 'agexcat' with Id  4
   Category 'agecat' with Id  5
   Category 'marrow10' with Id  7

   Total mean age at exposure [yr]  22.4097
   Total mean age attained [yr]  50.4871
   Total mean age of cases (approx.) [yr]  58.3279
   Total mean dose [Gy] 0.133906

-- Additional used categories
   none

-- Optimisation results
```

```
 Optimisation strategy 'medium'
 Number of model calls: 159
      Initial deviance:  2674.90962
        Final deviance:  2674.90962
             Reduction: 3.70649e−07

        Risk model 'elk_swcs'
Objective function 'poisson'
        Error mode 'minos'
    MINUIT2 errdef: 1
```

Model parameter

no.	name	unit	value	eparab	eminus	eplus	var/fix
0	b0	[−]	−9.51062	0.108478	−0.109504	0.106985	varbl
1	bsex	[−]	−0.321954	0.0571418	−0.0571957	0.0571152	varbl
2	bcity	[−]	−0.127091	0.0650619	−0.0657793	0.0644316	varbl
3	ba1	[−]	2.14537	0.272597	−0.272448	0.277593	varbl
4	ba2	[−]	1.05737	0.20938	−0.213776	0.203135	varbl
5	be1	[1/yr]	0.00661792	0.00458607	−0.00458527	0.00460542	varbl
6	be2	[1/yr^2]	−0.000734848	0.000227349	−0.000230586	0.000222978	varbl
7	os	[−]	0	−	−	−	fixed
8	err	[1/Gy]	1.37911	0.730497	−0.688486	0.790215	varbl
9	err2	[1/Gy^2]	1.3282	0.424152	−0.415872	0.443015	varbl
10	gam_e	[1/yr]	0	−	−	−	fixed
11	cen_e	[yr]	30	−	−	−	fixed
12	eps_a	[−]	−1.63408	0.366477	−0.385177	0.356668	varbl
13	cen_a	[yr]	55	−	−	−	fixed
14	nu_exp	[1/Gy]	0	−	−	−	fixed

Model parameter (final vs. initial)

no.	name	unit	final	initial	change	rel. ch. [%]
0	b0	[−]	−9.51062	−9.51061	−8.8842e−06	9.34135e−05
1	bsex	[−]	−0.321954	−0.321946	−7.58255e−06	0.00235522
2	bcity	[−]	−0.127091	−0.1271	8.81351e−06	−0.00693432
3	ba1	[−]	2.14537	2.14537	−2.38675e−06	−0.000111251
4	ba2	[−]	1.05737	1.05737	−4.30659e−06	−0.000407293
5	be1	[1/yr]	0.00661792	0.00661751	4.10253e−07	0.00619951
6	be2	[1/yr^2]	−0.000734848	−0.000734838	−1.00928e−08	0.00137347
7	os	[−]	fixed	0		
8	err	[1/Gy]	1.37911	1.37917	−5.6182e−05	−0.00407361
9	err2	[1/Gy^2]	1.3282	1.32824	−3.8731e−05	−0.00291597
10	gam_e	[1/yr]	fixed	0		
11	cen_e	[yr]	fixed	30		
12	eps_a	[−]	−1.63408	−1.63408	−5.92354e−07	3.625e−05
13	cen_a	[yr]	fixed	55		
14	nu_exp	[1/Gy]	fixed	0		

```
  Correlation matrix (of 10 variable parameters)
    0
    1   -0.050868613
    2     0.27521256   0.0084328087
    3     -0.3836162   -0.080576806    0.019391967
    4    -0.23189272   -0.049037666    0.015137025     0.33813065
    5     0.21512457    0.009790854    0.072645842    -0.51044751     -0.1704631
    6    -0.51520471     0.13490617   -0.017359916    0.094981939    -0.15511989    -0.11698794
    7    -0.36079863   -0.012473474     0.06950285      0.1634928   0.0064202555   -0.017453726    0.017786603
    8   -0.085138824     0.04858812    -0.03403968   -0.023324548       0.112168    0.019827804    0.022920313    -0.62777981
    9   -0.063671778    0.026180341  -0.0067275759    -0.38630651     0.55878077    0.019561527   0.0021218262  -0.0062184377
     0.18557031

-- O/E analysis
   Checking sums for persons, person years and cases
                          Stratification   O/E analysis
          Total persons:           86611          86611
     Total person years:     3.29428e+06    3.29428e+06
   Total observed cases:             318            318
   Total expected cases:         317.998        317.998
               Deviance:         2674.91        40.6467

   Cases
     Excess:     99.7
   Baseline:    218.3
      Total:    318.0

   Categories
        name     unit  id  size  used
     agexcat       yr   4     4     4
      agecat       yr   5     4     4
    marrow10       Gy   7     5     4

   Number of Poisson cells
                 Stratification   O/E analysis
   Total cells:           27720             64
    Used cells:           53782             40

   Poisson cells
   cell subjects         pyr  cases      obs haz    ubnd 4      mean 4    ubnd 5      mean 5    ubnd 7      mean 7
      0    13057   101123.29      1  9.88892e-06        20     4.46854        20     14.8023       0.5   0.0939403
      1     3975   324053.35     15  4.62887e-05        20      9.1773        40     30.1791       0.5   0.0975599
      2     8104    57092.47      3  5.25463e-05        40     25.0347        40     35.0642       0.5    0.107059
      3        0   315836.96     12  3.79943e-05        20     9.39005        60     49.8374       0.5   0.0979589
      4     3305   205804.81      6  2.91538e-05        40     30.2796        60     50.1692       0.5    0.107652
      5     9067    68352.41      6  8.77804e-05        60     44.8118        60     54.8787       0.5    0.109273
      6        0   109490.15     13  0.000118732        20     13.5791     infty     65.7873       0.5     0.10176
```

7	0	209170.81	37	0.000176889	40	30.8783	infty	72.0007	0.5	0.107023
8	1997	191482.30	28	0.000146228	60	48.5002	infty	72.2454	0.5	0.108687
9	2786	27718.45	2	7.21541e-05	infty	65.2003	infty	77.626	0.5	0.102219
10	1207	8215.25	8	0.000973799	20	4.88333	20	15.0014	1.5	0.978151
11	737	36059.42	8	0.000221856	20	11.1884	40	30.3333	1.5	0.97297
12	969	7463.32	1	0.000133989	40	24.5702	40	34.8287	1.5	0.977193
13	0	35265.68	8	0.000226849	20	11.4733	60	49.8035	1.5	0.97049
14	378	24001.33	7	0.00029165	40	29.6972	60	50.1327	1.5	0.984573
15	1027	7870.86	2	0.000254102	60	44.6862	60	54.8289	1.5	0.967113
16	0	15045.50	4	0.00026586	20	14.8263	infty	66.3623	1.5	0.967312
17	0	22505.23	8	0.000355473	40	30.2714	infty	71.4529	1.5	0.982348
18	185	19764.19	11	0.000556562	60	48.1545	infty	71.7338	1.5	0.952927
19	195	1922.04	0	0	infty	64.4784	infty	77.0941	1.5	0.912676
20	348	2263.04	6	0.0026513	20	4.80715	20	14.9324	infty	2.60704
21	175	9511.55	5	0.000525677	20	11.1083	40	30.3187	infty	2.52412
22	226	1763.85	2	0.00113389	40	24.4504	40	34.77	infty	2.43736
23	0	8996.04	2	0.00022232	20	11.4226	60	49.6696	infty	2.51478
24	76	5225.31	8	0.00153101	40	29.2529	60	49.9794	infty	2.43729
25	209	1641.38	2	0.00121849	60	44.6789	60	54.7877	infty	2.43315
26	0	3341.08	4	0.00119722	20	14.7015	infty	66.0517	infty	2.46649
27	0	4238.19	5	0.00117975	40	29.4826	infty	70.5257	infty	2.42636
28	37	3425.07	4	0.00116786	60	47.9354	infty	70.7562	infty	2.46001
29	42	369.32	1	0.00270768	infty	63.679	infty	75.8466	infty	2.51524

Observed/expected comparison

cell	pyr			cases			hazard	cell dev.
		obs	exp	bsl	obs	exp	bsl	
0	101123.29	1	5.1	1.8	9.88892e-06	4.99918e-05	1.78453e-05	4.86978
1	324053.35	15	10.3	7.0	4.62887e-05	3.18454e-05	2.16683e-05	1.85934
2	57092.47	3	2.5	1.8	5.25463e-05	4.38931e-05	3.14389e-05	0.0915607
3	315836.96	12	16.5	13.7	3.79943e-05	5.20891e-05	4.34024e-05	1.33085
4	205804.81	6	15.0	12.3	2.91538e-05	7.29763e-05	5.97537e-05	7.02718
5	68352.41	6	6.1	5.1	8.77804e-05	8.9208e-05	7.45764e-05	0.00157001
6	109490.15	13	11.1	9.8	0.000118732	0.000101293	8.95126e-05	0.311326
7	209170.81	37	32.2	28.7	0.000176889	0.000153809	0.000137338	0.69068
8	191482.30	28	29.5	26.3	0.000146228	0.000154139	0.000137446	0.0791196
9	27718.45	2	2.9	2.7	7.21541e-05	0.000105984	9.60271e-05	0.337493
10	8215.25	8	4.6	0.1	0.000973799	0.000557247	1.75521e-05	2.08698
11	36059.42	8	6.9	0.8	0.000221856	0.000191516	2.17589e-05	0.164835
12	7463.32	1	1.6	0.2	0.000133989	0.000218006	3.04774e-05	0.28056
13	35265.68	8	6.7	1.5	0.000226849	0.000188767	4.35803e-05	0.254363
14	24001.33	7	6.4	1.4	0.00029165	0.000264726	6.01412e-05	0.0636023
15	7870.86	2	2.4	0.6	0.000254102	0.000301851	7.72861e-05	0.0628615
16	15045.50	4	4.0	1.3	0.00026586	0.00026895	8.76225e-05	0.000536193
17	22505.23	8	8.8	3.0	0.000355473	0.000390082	0.000135073	0.0712455
18	19764.19	11	7.7	2.7	0.000556562	0.000388914	0.000138731	1.25839
19	1922.04	0	0.5	0.2	0	0.000248904	9.97069e-05	0.956805

20	2263.04	6	6.3	0.0	0.0026513	0.00276618	1.74948e−05	0.0109499
21	9511.55	5	8.3	0.2	0.000525677	0.000870551	2.36465e−05	1.51618
22	1763.85	2	1.5	0.1	0.00113389	0.00084167	3.07014e−05	0.161224
23	8996.04	2	6.9	0.4	0.00022232	0.000771848	4.73422e−05	4.90847
24	5225.31	8	4.8	0.3	0.00153101	0.000927932	5.94821e−05	1.70905
25	1641.38	2	1.8	0.1	0.00121849	0.00106663	7.70308e−05	0.0339131
26	3341.08	4	3.4	0.3	0.00119722	0.00101299	9.83889e−05	0.105713
27	4238.19	5	5.0	0.6	0.00117975	0.00118926	0.000130342	0.000323517
28	3425.07	4	4.4	0.5	0.00116786	0.00128826	0.000136223	0.0397986
29	369.32	1	0.4	0.0	0.00270768	0.00103673	0.00011799	0.685814
