# Supplementary material for: Independent analysis of the radiation risk for leukaemia in children and adults with mortality data (1950–2003) of Japanese A-bomb survivors
Source: Radiat Environ Biophys. 2012 Nov 4;52(1):17–27. doi: 10.1007/s00411-012-0437-6 (PMC3579470; doi:10.1007/s00411-012-0437-6)
Supplement: Supplementary file 3 — PDF (36 KB) [file 411_2012_437_MOESM3_ESM.pdf]

```
-- Result protocol
   From MECAN version 0.2
   Based on MINUIT2 version 5.27.02
   In file format version rec.1.0
   Parallelisation mode 'useOpenmpDefault'
   Operation mode 'regression & analysis'
   Group data mode 'use_raw_data'

-- Control files
   MINUIT2 file 'qexp.min'
   Control file 'par/leuk.par'

-- Optimisation time
   Optimisation started: Mi. Aug 15 13:57:32 2012
   Optimisation stopped: Mi. Aug 15 14:05:36 2012
           Elapsed time: 00:08:04 or 484 secs

-- Raw data summary
   Grouped data read from file 'lss14.csv'
   Created 53782 Poisson cells with 8 categories
     for organ/organ group 'leukemia'
   Counted          86611 persons
   Counted      3294282.3 person years
   Counted            318 cases
   Case counts pertain to end point(s) for 1 organ/organ groups
   End point no.  1 'Leukemia 204-208'
   Organ dose 'marrow10' with Id 9

-- Stratification summary
   Created 53782 Poisson cells with 5 categories
     for organ/organ group 'leukemia'
   Category  'city' with Id  0
   Category   'sex' with Id  1
   Category 'agexcat' with Id  4
   Category 'agecat' with Id  5
   Category 'marrow10' with Id  7

   Total mean age at exposure [yr]  22.4097
   Total mean age attained [yr]  50.4871
   Total mean age of cases (approx.) [yr]  58.3279
   Total mean dose [Gy] 0.133906

-- Additional used categories
   none

-- Optimisation results
```

```
 Optimisation strategy 'medium'
 Number of model calls: 149
      Initial deviance:  2670.88980
        Final deviance:  2670.88980
             Reduction: 5.96277e−07

        Risk model 'elk_swcs'
Objective function 'poisson'
        Error mode 'minos'
    MINUIT2 errdef: 1
```

Model parameter

| no. | name | unit | value | eparab | eminus | eplus | var/fix |
|---|---|---|---|---|---|---|---|
| 0 | b0 | [−] | −9.49592 | 0.104876 | −0.105789 | 0.10354 | varbl |
| 1 | bsex | [−] | −0.322168 | 0.0571363 | −0.0571745 | 0.057124 | varbl |
| 2 | bcity | [−] | −0.140183 | 0.0649278 | −0.0656532 | 0.0642816 | varbl |
| 3 | ba1 | [−] | 2.11138 | 0.26537 | −0.26536 | 0.269033 | varbl |
| 4 | ba2 | [−] | 1.07978 | 0.205344 | −0.209542 | 0.199365 | varbl |
| 5 | be1 | [1/yr] | 0.00640716 | 0.00458611 | −0.00458636 | 0.00460414 | varbl |
| 6 | be2 | [1/yr^2] | −0.00071956 | 0.000227255 | −0.000230483 | 0.000222893 | varbl |
| 7 | os | [−] | 0 | − | − | − | fixed |
| 8 | err | [1/Gy] | 0 | − | − | − | fixed |
| 9 | err2 | [1/Gy^2] | 4.2884 | 1.24178 | −1.09762 | 1.38497 | varbl |
| 10 | gam_e | [1/yr] | 0 | − | − | − | fixed |
| 11 | cen_e | [yr] | 30 | − | − | − | fixed |
| 12 | eps_a | [−] | −1.61701 | 0.346829 | −0.363812 | 0.337504 | varbl |
| 13 | cen_a | [yr] | 55 | − | − | − | fixed |
| 14 | nu_exp | [1/Gy] | −0.378203 | 0.128305 | −0.12754 | 0.127259 | varbl |

Model parameter (final vs. initial)

| no. | name | unit | final | initial | change | rel. ch. [%] |
|---|---|---|---|---|---|---|
| 0 | b0 | [−] | −9.49592 | −9.49592 | 4.25447e−07 | −4.48031e−06 |
| 1 | bsex | [−] | −0.322168 | −0.322157 | −1.08129e−05 | 0.00335639 |
| 2 | bcity | [−] | −0.140183 | −0.140184 | 5.03793e−07 | −0.00035938 |
| 3 | ba1 | [−] | 2.11138 | 2.11139 | −1.31724e−05 | −0.000623872 |
| 4 | ba2 | [−] | 1.07978 | 1.07979 | −1.34788e−05 | −0.00124828 |
| 5 | be1 | [1/yr] | 0.00640716 | 0.00640753 | −3.70639e−07 | −0.00578443 |
| 6 | be2 | [1/yr^2] | −0.00071956 | −0.000719578 | 1.84582e−08 | −0.00256514 |
| 7 | os | [−] | fixed | 0 | | |
| 8 | err | [1/Gy] | fixed | 0 | | |
| 9 | err2 | [1/Gy^2] | 4.2884 | 4.28823 | 0.000169738 | 0.00395824 |
| 10 | gam_e | [1/yr] | fixed | 0 | | |
| 11 | cen_e | [yr] | fixed | 30 | | |
| 12 | eps_a | [−] | −1.61701 | −1.61691 | −9.61698e−05 | 0.00594775 |
| 13 | cen_a | [yr] | fixed | 55 | | |
| 14 | nu_exp | [1/Gy] | −0.378203 | −0.378183 | −2.01428e−05 | 0.00532621 |

```
  Correlation matrix (of 10 variable parameters)
    0
    1   -0.054199075
    2     0.30999583    0.010890369
    3    -0.37206068   -0.079637049   0.0094247507
    4    -0.24654291   -0.051030509    0.017582308     0.39606974
    5     0.22399732   0.0090184885    0.074664875    -0.52349315    -0.17368283
    6    -0.53432929     0.13505065   -0.017312025    0.098552516     -0.1598525    -0.11907908
    7    -0.36695208   0.0049843829   0.0026283116     0.13421981    0.057827306     -0.0207253     0.04366759
    8   -0.079690886    0.024461571  -0.0015639998    -0.34341765     0.53262695    0.021427128 -0.00029790086     0.07036771
    9     0.11418096    0.024424399   0.0071096078   -0.066405853    0.028162094    0.026113252   -0.016307759    -0.82046297
    0.080954282

-- O/E analysis
   Checking sums for persons, person years and cases
                          Stratification   O/E analysis
         Total persons:            86611          86611
     Total person years:     3.29428e+06    3.29428e+06
   Total observed cases:             318            318
   Total expected cases:         318.004        318.004
               Deviance:         2670.89        37.2576

   Cases
     Excess:     94.3
   Baseline:    223.7
      Total:    318.0

   Categories
        name     unit  id  size  used
     agexcat       yr   4     4     4
      agecat       yr   5     4     4
    marrow10       Gy   7     5     4

   Number of Poisson cells
                 Stratification   O/E analysis
   Total cells:           27720             64
    Used cells:           53782             40

   Poisson cells
   cell subjects         pyr  cases      obs haz    ubnd 4      mean 4    ubnd 5      mean 5    ubnd 7      mean 7
      0    13057   101123.29      1  9.88892e-06        20     4.46854        20     14.8023       0.5   0.0939403
      1     3975   324053.35     15  4.62887e-05        20      9.1773        40     30.1791       0.5   0.0975599
      2     8104    57092.47      3  5.25463e-05        40     25.0347        40     35.0642       0.5    0.107059
      3        0   315836.96     12  3.79943e-05        20     9.39005        60     49.8374       0.5   0.0979589
      4     3305   205804.81      6  2.91538e-05        40     30.2796        60     50.1692       0.5    0.107652
      5     9067    68352.41      6  8.77804e-05        60     44.8118        60     54.8787       0.5    0.109273
      6        0   109490.15     13  0.000118732        20     13.5791     infty     65.7873       0.5     0.10176
```

| | | | | | | | | | | |
|---|---|---|---|---|---|---|---|---|---|---|
| 7 | 0 | 209170.81 | 37 | 0.000176889 | 40 | 30.8783 | infty | 72.0007 | 0.5 | 0.107023 |
| 8 | 1997 | 191482.30 | 28 | 0.000146228 | 60 | 48.5002 | infty | 72.2454 | 0.5 | 0.108687 |
| 9 | 2786 | 27718.45 | 2 | 7.21541e-05 | infty | 65.2003 | infty | 77.626 | 0.5 | 0.102219 |
| 10 | 1207 | 8215.25 | 8 | 0.000973799 | 20 | 4.88333 | 20 | 15.0014 | 1.5 | 0.978151 |
| 11 | 737 | 36059.42 | 8 | 0.000221856 | 20 | 11.1884 | 40 | 30.3333 | 1.5 | 0.97297 |
| 12 | 969 | 7463.32 | 1 | 0.000133989 | 40 | 24.5702 | 40 | 34.8287 | 1.5 | 0.977193 |
| 13 | 0 | 35265.68 | 8 | 0.000226849 | 20 | 11.4733 | 60 | 49.8035 | 1.5 | 0.97049 |
| 14 | 378 | 24001.33 | 7 | 0.00029165 | 40 | 29.6972 | 60 | 50.1327 | 1.5 | 0.984573 |
| 15 | 1027 | 7870.86 | 2 | 0.000254102 | 60 | 44.6862 | 60 | 54.8289 | 1.5 | 0.967113 |
| 16 | 0 | 15045.50 | 4 | 0.00026586 | 20 | 14.8263 | infty | 66.3623 | 1.5 | 0.967312 |
| 17 | 0 | 22505.23 | 8 | 0.000355473 | 40 | 30.2714 | infty | 71.4529 | 1.5 | 0.982348 |
| 18 | 185 | 19764.19 | 11 | 0.000556562 | 60 | 48.1545 | infty | 71.7338 | 1.5 | 0.952927 |
| 19 | 195 | 1922.04 | 0 | 0 | infty | 64.4784 | infty | 77.0941 | 1.5 | 0.912676 |
| 20 | 348 | 2263.04 | 6 | 0.0026513 | 20 | 4.80715 | 20 | 14.9324 | infty | 2.60704 |
| 21 | 175 | 9511.55 | 5 | 0.000525677 | 20 | 11.1083 | 40 | 30.3187 | infty | 2.52412 |
| 22 | 226 | 1763.85 | 2 | 0.00113389 | 40 | 24.4504 | 40 | 34.77 | infty | 2.43736 |
| 23 | 0 | 8996.04 | 2 | 0.00022232 | 20 | 11.4226 | 60 | 49.6696 | infty | 2.51478 |
| 24 | 76 | 5225.31 | 8 | 0.00153101 | 40 | 29.2529 | 60 | 49.9794 | infty | 2.43729 |
| 25 | 209 | 1641.38 | 2 | 0.00121849 | 60 | 44.6789 | 60 | 54.7877 | infty | 2.43315 |
| 26 | 0 | 3341.08 | 4 | 0.00119722 | 20 | 14.7015 | infty | 66.0517 | infty | 2.46649 |
| 27 | 0 | 4238.19 | 5 | 0.00117975 | 40 | 29.4826 | infty | 70.5257 | infty | 2.42636 |
| 28 | 37 | 3425.07 | 4 | 0.00116786 | 60 | 47.9354 | infty | 70.7562 | infty | 2.46001 |
| 29 | 42 | 369.32 | 1 | 0.00270768 | infty | 63.679 | infty | 75.8466 | infty | 2.51524 |

Observed/expected comparison

| cell | pyr | cases obs | cases exp | cases bsl | hazard obs | hazard exp | hazard bsl | cell dev. |
|---|---|---|---|---|---|---|---|---|
| 0 | 101123.29 | 1 | 4.3 | 2.1 | 9.88892e-06 | 4.22226e-05 | 2.03329e-05 | 3.6363 |
| 1 | 324053.35 | 15 | 9.6 | 7.5 | 4.62887e-05 | 2.95069e-05 | 2.30393e-05 | 2.6318 |
| 2 | 57092.47 | 3 | 2.3 | 1.9 | 5.25463e-05 | 4.10205e-05 | 3.28698e-05 | 0.169663 |
| 3 | 315836.96 | 12 | 15.9 | 14.2 | 3.79943e-05 | 5.04192e-05 | 4.49874e-05 | 1.05803 |
| 4 | 205804.81 | 6 | 14.4 | 12.6 | 2.91538e-05 | 6.99601e-05 | 6.13937e-05 | 6.29218 |
| 5 | 68352.41 | 6 | 5.9 | 5.2 | 8.77804e-05 | 8.58824e-05 | 7.64057e-05 | 0.00284617 |
| 6 | 109490.15 | 13 | 10.8 | 10.0 | 0.000118732 | 9.90815e-05 | 9.17002e-05 | 0.401005 |
| 7 | 209170.81 | 37 | 31.4 | 29.2 | 0.000176889 | 0.000150153 | 0.000139595 | 0.94144 |
| 8 | 191482.30 | 28 | 28.9 | 26.8 | 0.000146228 | 0.00015073 | 0.000139921 | 0.0260118 |
| 9 | 27718.45 | 2 | 2.9 | 2.7 | 7.21541e-05 | 0.000104925 | 9.86333e-05 | 0.318946 |
| 10 | 8215.25 | 8 | 5.7 | 0.2 | 0.000973799 | 0.000697822 | 1.99378e-05 | 0.797415 |
| 11 | 36059.42 | 8 | 8.0 | 0.8 | 0.000221856 | 0.000222611 | 2.29958e-05 | 9.25249e-05 |
| 12 | 7463.32 | 1 | 1.9 | 0.2 | 0.000133989 | 0.000248751 | 3.17629e-05 | 0.47562 |
| 13 | 35265.68 | 8 | 7.5 | 1.6 | 0.000226849 | 0.000212443 | 4.49111e-05 | 0.0337012 |
| 14 | 24001.33 | 7 | 7.1 | 1.5 | 0.00029165 | 0.00029585 | 6.16254e-05 | 0.00143773 |
| 15 | 7870.86 | 2 | 2.6 | 0.6 | 0.000254102 | 0.000336508 | 7.91247e-05 | 0.173669 |
| 16 | 15045.50 | 4 | 4.5 | 1.3 | 0.00026586 | 0.000296843 | 8.91527e-05 | 0.0504406 |
| 17 | 22505.23 | 8 | 9.6 | 3.1 | 0.000355473 | 0.000427453 | 0.000136986 | 0.289533 |
| 18 | 19764.19 | 11 | 8.4 | 2.8 | 0.000556562 | 0.000427204 | 0.000141144 | 0.706065 |
| 19 | 1922.04 | 0 | 0.5 | 0.2 | 0 | 0.000272485 | 0.000102352 | 1.04745 |

| | | | | | | | | |
|---|---|---|---|---|---|---|---|---|
| 20 | 2263.04 | 6 | 5.7 | 0.0 | 0.0026513 | 0.00253225 | 1.98599e−05 | 0.0124709 |
| 21 | 9511.55 | 5 | 7.6 | 0.2 | 0.000525677 | 0.000797827 | 2.50546e−05 | 1.00509 |
| 22 | 1763.85 | 2 | 1.4 | 0.1 | 0.00113389 | 0.000782906 | 3.20447e−05 | 0.243425 |
| 23 | 8996.04 | 2 | 6.3 | 0.4 | 0.00022232 | 0.000701169 | 4.89302e−05 | 4.02096 |
| 24 | 5225.31 | 8 | 4.4 | 0.3 | 0.00153101 | 0.000850436 | 6.09497e−05 | 2.29452 |
| 25 | 1641.38 | 2 | 1.6 | 0.1 | 0.00121849 | 0.000975458 | 7.88496e−05 | 0.0920274 |
| 26 | 3341.08 | 4 | 3.1 | 0.3 | 0.00119722 | 0.000934834 | 0.000100631 | 0.225803 |
| 27 | 4238.19 | 5 | 4.6 | 0.6 | 0.00117975 | 0.0010949 | 0.000132045 | 0.0271751 |
| 28 | 3425.07 | 4 | 4.0 | 0.5 | 0.00116786 | 0.00116979 | 0.000138598 | 1.09794e−05 |
| 29 | 369.32 | 1 | 0.3 | 0.0 | 0.00270768 | 0.000946093 | 0.00012134 | 0.801836 |
