# Supplementary material for: Independent analysis of the radiation risk for leukaemia in children and adults with mortality data (1950–2003) of Japanese A-bomb survivors
Source: Radiat Environ Biophys. 2012 Nov 4;52(1):17–27. doi: 10.1007/s00411-012-0437-6 (PMC3579470; doi:10.1007/s00411-012-0437-6)
Supplement: Supplementary file 4 — PDF (36 KB) [file 411_2012_437_MOESM4_ESM.pdf]

```
-- Result protocol
   From MECAN version 0.2
   Based on MINUIT2 version 5.27.02
   In file format version rec.1.0
   Parallelisation mode ’useOpenmpDefault’
   Operation mode ’regression & analysis’
   Group data mode ’use_raw_data’

-- Control files
   MINUIT2 file ’sigmoid.min’
   Control file ’par/leuk.par’

-- Optimisation time
   Optimisation started: Mi. Aug 15 14:39:09 2012
   Optimisation stopped: Mi. Aug 15 14:52:32 2012
           Elapsed time: 00:13:22 or 802 secs

-- Raw data summary
   Grouped data read from file ’lss14.csv’
   Created 53782 Poisson cells with 8 categories
     for organ/organ group ’leukemia’
   Counted          86611 persons
   Counted      3294282.3 person years
   Counted            318 cases
   Case counts pertain to end point(s) for 1 organ/organ groups
   End point no.  1 ’Leukemia 204−208’
   Organ dose ’marrow10’ with Id 9

-- Stratification summary
   Created 53782 Poisson cells with 5 categories
     for organ/organ group ’leukemia’
   Category  ’city’ with Id  0
   Category   ’sex’ with Id  1
   Category ’agexcat’ with Id  4
   Category ’agecat’ with Id  5
   Category ’marrow10’ with Id  7

   Total mean age at exposure [yr]  22.4097
   Total mean age attained [yr]  50.4871
   Total mean age of cases (approx.) [yr]  58.3279
   Total mean dose [Gy] 0.133906

-- Additional used categories
   none

-- Optimisation results
```

```
 Optimisation strategy 'medium'
 Number of model calls: 169
      Initial deviance:  2670.77787
        Final deviance:  2670.77787
             Reduction: 5.84481e−06

        Risk model 'elk_sigmoid_err'
Objective function 'poisson'
        Error mode 'minos'
    MINUIT2 errdef: 1
```

Model parameter

no.	name	unit	value	eparab	eminus	eplus	var/fix	logtrans
0	b0	[−]	−9.49135	0.105473	−0.106413	0.104193	varbl	−
1	bsex	[−]	−0.321779	0.0571472	−0.0572087	0.0571117	varbl	−
2	bcity	[−]	−0.142683	0.0653611	−0.0660621	0.0647581	varbl	−
3	ba1	[−]	2.1012	0.265888	−0.265139	0.27049	varbl	−
4	ba2	[−]	1.08448	0.205288	−0.209822	0.199077	varbl	−
5	be1	[1/yr]	0.00640818	0.00458583	−0.00459153	0.00459849	varbl	−
6	be2	[1/yr^2]	−0.000717607	0.000227255	−0.000230825	0.000222595	varbl	−
7	B	[−]	−1.67301	0.873029	−1.45344	0.738449	varbl	log
8	C	[−]	0.71889	0.259234	−0.254308	0.266312	varbl	log
9	A	[1/Gy]	3.9354	1.15729	−0.973022	1.44146	varbl	−
10	pa	[−]	−1.60747	0.344396	−0.363427	0.33374	varbl	−
11	cen_a	[yr]	55	−	−	−	fixed	−
12	px	[1/yr]	0	−	−	−	fixed	−
13	cen_e	[yr]	30	−	−	−	fixed	−
14	msex	[−]	0	−	−	−	fixed	−

Model parameter (final vs. initial)

no.	name	unit	final	initial	change	rel. ch. [%]
0	b0	[−]	−9.49135	−9.49138	3.26371e−05	−0.000343861
1	bsex	[−]	−0.321779	−0.321775	−3.97722e−06	0.00123603
2	bcity	[−]	−0.142683	−0.142664	−1.85773e−05	0.0130217
3	ba1	[−]	2.1012	2.10121	−5.11886e−06	−0.000243615
4	ba2	[−]	1.08448	1.08446	1.93527e−05	0.00178455
5	be1	[1/yr]	0.00640818	0.00640958	−1.40434e−06	−0.0219101
6	be2	[1/yr^2]	−0.000717607	−0.000717631	2.43155e−08	−0.00338831
7	B	[−]	−1.67301	−1.67284	−0.000171094	0.0102278
8	C	[−]	0.71889	0.718865	2.47481e−05	0.00344266
9	A	[1/Gy]	3.9354	3.93519	0.000207338	0.00526882
10	pa	[−]	−1.60747	−1.60738	−9.24087e−05	0.00574903
11	cen_a	[yr]	fixed	55		
12	px	[1/yr]	fixed	0		
13	cen_e	[yr]	fixed	30		
14	msex	[−]	fixed	0		

```
  Correlation matrix (of 11 variable parameters)
    0
    1   −0.051936362
    2     0.29234689   0.0088647651
    3    −0.38103917    −0.08096417    0.022293151
    4     −0.2329433   −0.049607672    0.007401158     0.39343666
    5     0.22133698   0.0087541277    0.074900179    −0.52123065    −0.17430951
    6    −0.52758393     0.13545952   −0.019806178    0.095353334     −0.1579745    −0.11931977
    7    0.041188378  −0.0025881854   −0.094555972   −0.053148358    0.046191385   −0.020112049    0.024921892
    8     0.13835474    0.016550779    −0.11473124    −0.11648001    0.085003016  −0.0067090814    0.022022683     0.77481407
    9    −0.27815138    0.014918316   −0.049372858    0.068264103    0.093734749   −0.018831014    0.048540709     0.77955197
     0.42943541
   10   −0.069359846    0.025844934   −0.012590688    −0.34049766     0.53130867    0.020616121   0.0022440142    0.026504254
    0.094185375     0.12543765

-- O/E analysis
   Checking sums for persons, person years and cases
                         Stratification   O/E analysis
          Total persons:          86611          86611
     Total person years:    3.29428e+06    3.29428e+06
   Total observed cases:            318            318
   Total expected cases:        317.993        317.993
               Deviance:        2670.78        36.8819

   Cases
     Excess:     92.9
   Baseline:    225.1
      Total:    318.0

   Categories
        name     unit  id  size  used
     agexcat       yr   4     4     4
      agecat       yr   5     4     4
    marrow10       Gy   7     5     4

   Number of Poisson cells
                 Stratification   O/E analysis
   Total cells:           27720             64
    Used cells:           53782             40

   Poisson cells
   cell subjects         pyr  cases      obs haz    ubnd 4      mean 4    ubnd 5      mean 5    ubnd 7      mean 7
      0    13057   101123.29      1  9.88892e-06        20     4.46854        20     14.8023       0.5   0.0939403
      1     3975   324053.35     15  4.62887e-05        20      9.1773        40     30.1791       0.5   0.0975599
      2     8104    57092.47      3  5.25463e-05        40     25.0347        40     35.0642       0.5    0.107059
      3        0   315836.96     12  3.79943e-05        20     9.39005        60     49.8374       0.5   0.0979589
      4     3305   205804.81      6  2.91538e-05        40     30.2796        60     50.1692       0.5    0.107652
```

5	9067	68352.41	6	8.77804e-05	60	44.8118	60	54.8787	0.5	0.109273
6	0	109490.15	13	0.000118732	20	13.5791	infty	65.7873	0.5	0.10176
7	0	209170.81	37	0.000176889	40	30.8783	infty	72.0007	0.5	0.107023
8	1997	191482.30	28	0.000146228	60	48.5002	infty	72.2454	0.5	0.108687
9	2786	27718.45	2	7.21541e-05	infty	65.2003	infty	77.626	0.5	0.102219
10	1207	8215.25	8	0.000973799	20	4.88333	20	15.0014	1.5	0.978151
11	737	36059.42	8	0.000221856	20	11.1884	40	30.3333	1.5	0.97297
12	969	7463.32	1	0.000133989	40	24.5702	40	34.8287	1.5	0.977193
13	0	35265.68	8	0.000226849	20	11.4733	60	49.8035	1.5	0.97049
14	378	24001.33	7	0.00029165	40	29.6972	60	50.1327	1.5	0.984573
15	1027	7870.86	2	0.000254102	60	44.6862	60	54.8289	1.5	0.967113
16	0	15045.50	4	0.00026586	20	14.8263	infty	66.3623	1.5	0.967312
17	0	22505.23	8	0.000355473	40	30.2714	infty	71.4529	1.5	0.982348
18	185	19764.19	11	0.000556562	60	48.1545	infty	71.7338	1.5	0.952927
19	195	1922.04	0	0	infty	64.4784	infty	77.0941	1.5	0.912676
20	348	2263.04	6	0.0026513	20	4.80715	20	14.9324	infty	2.60704
21	175	9511.55	5	0.000525677	20	11.1083	40	30.3187	infty	2.52412
22	226	1763.85	2	0.00113389	40	24.4504	40	34.77	infty	2.43736
23	0	8996.04	2	0.00022232	20	11.4226	60	49.6696	infty	2.51478
24	76	5225.31	8	0.00153101	40	29.2529	60	49.9794	infty	2.43729
25	209	1641.38	2	0.00121849	60	44.6789	60	54.7877	infty	2.43315
26	0	3341.08	4	0.00119722	20	14.7015	infty	66.0517	infty	2.46649
27	0	4238.19	5	0.00117975	40	29.4826	infty	70.5257	infty	2.42636
28	37	3425.07	4	0.00116786	60	47.9354	infty	70.7562	infty	2.46001
29	42	369.32	1	0.00270768	infty	63.679	infty	75.8466	infty	2.51524

Observed/expected comparison

cell	pyr			cases			hazard	cell dev.
		obs	exp	bsl	obs	exp	bsl	
0	101123.29	1	4.1	2.1	9.88892e-06	4.01147e-05	2.09731e-05	3.3124
1	324053.35	15	9.4	7.6	4.62887e-05	2.89708e-05	2.3376e-05	2.83443
2	57092.47	3	2.3	1.9	5.25463e-05	4.03502e-05	3.32632e-05	0.191977
3	315836.96	12	15.8	14.3	3.79943e-05	5.00217e-05	4.53317e-05	0.996889
4	205804.81	6	14.3	12.7	2.91538e-05	6.92806e-05	6.18449e-05	6.12963
5	68352.41	6	5.8	5.3	8.77804e-05	8.5149e-05	7.69215e-05	0.00550185
6	109490.15	13	10.8	10.1	0.000118732	9.85171e-05	9.21366e-05	0.425943
7	209170.81	37	31.2	29.3	0.000176889	0.000149317	0.000140157	1.00488
8	191482.30	28	28.7	26.9	0.000146228	0.000149952	0.000140552	0.0178625
9	27718.45	2	2.9	2.8	7.21541e-05	0.000104663	9.92141e-05	0.314435
10	8215.25	8	5.9	0.2	0.000973799	0.000715764	2.05526e-05	0.686027
11	36059.42	8	8.1	0.8	0.000221856	0.000225648	2.33087e-05	0.00231115
12	7463.32	1	1.9	0.2	0.000133989	0.000251941	3.2123e-05	0.497752
13	35265.68	8	7.6	1.6	0.000226849	0.000214596	4.52133e-05	0.0242178
14	24001.33	7	7.2	1.5	0.00029165	0.000299185	6.20422e-05	0.00459259
15	7870.86	2	2.7	0.6	0.000254102	0.00034013	7.96439e-05	0.187862
16	15045.50	4	4.5	1.3	0.00026586	0.000299254	8.94677e-05	0.0582754
17	22505.23	8	9.7	3.1	0.000355473	0.000431606	0.000137473	0.321761

18	19764.19	11	8.5	2.8	0.000556562	0.000431122	0.000141764	0.660088
19	1922.04	0	0.5	0.2	0	0.000274252	0.000102944	1.05425
20	2263.04	6	5.7	0.0	0.0026513	0.00253921	2.04706e−05	0.0110364
21	9511.55	5	7.6	0.2	0.000525677	0.000795274	2.54066e−05	0.98857
22	1763.85	2	1.4	0.1	0.00113389	0.00078193	3.24175e−05	0.24497
23	8996.04	2	6.3	0.4	0.00022232	0.00069804	4.92852e−05	3.98255
24	5225.31	8	4.4	0.3	0.00153101	0.000847655	6.13612e−05	2.31786
25	1641.38	2	1.6	0.1	0.00121849	0.000972553	7.93646e−05	0.094421
26	3341.08	4	3.1	0.3	0.00119722	0.000932126	0.000101079	0.230916
27	4238.19	5	4.6	0.6	0.00117975	0.00109228	0.000132485	0.0289239
28	3425.07	4	4.0	0.5	0.00116786	0.00116531	0.000139211	1.90489e−05
29	369.32	1	0.3	0.0	0.00270768	0.000944079	0.000122068	0.80461
