# Supplementary material for: Independent analysis of the radiation risk for leukaemia in children and adults with mortality data (1950–2003) of Japanese A-bomb survivors
Source: Radiat Environ Biophys. 2012 Nov 4;52(1):17–27. doi: 10.1007/s00411-012-0437-6 (PMC3579470; doi:10.1007/s00411-012-0437-6)
Supplement: Supplementary file 5 — PDF (35 KB) [file 411_2012_437_MOESM5_ESM.pdf]

```
-- Result protocol
   From MECAN version 0.2
   Based on MINUIT2 version 5.27.02
   In file format version rec.1.0
   Parallelisation mode ’useOpenmpDefault’
   Operation mode ’regression & analysis’
   Group data mode ’use_raw_data’

-- Control files
   MINUIT2 file ’spline.min’
   Control file ’par/leuk.par’

-- Optimisation time
   Optimisation started: Mi. Aug 15 14:52:39 2012
   Optimisation stopped: Mi. Aug 15 15:04:50 2012
           Elapsed time: 00:12:11 or 731 secs

-- Raw data summary
   Grouped data read from file ’lss14.csv’
   Created 53782 Poisson cells with 8 categories
     for organ/organ group ’leukemia’
   Counted          86611 persons
   Counted      3294282.3 person years
   Counted            318 cases
   Case counts pertain to end point(s) for 1 organ/organ groups
   End point no.  1 ’Leukemia 204−208’
   Organ dose ’marrow10’ with Id 9

-- Stratification summary
   Created 53782 Poisson cells with 5 categories
     for organ/organ group ’leukemia’
   Category  ’city’ with Id  0
   Category   ’sex’ with Id  1
   Category ’agexcat’ with Id  4
   Category ’agecat’ with Id  5
   Category ’marrow10’ with Id  7

   Total mean age at exposure [yr]  22.4097
   Total mean age attained [yr]  50.4871
   Total mean age of cases (approx.) [yr]  58.3279
   Total mean dose [Gy] 0.133906

-- Additional used categories
   none

-- Optimisation results
```

```
  Optimisation strategy 'medium'
  Number of model calls: 171
       Initial deviance:  2670.91385
         Final deviance:  2670.91385
              Reduction: 3.71096e-06

         Risk model 'elk_kink_err'
 Objective function 'poisson'
         Error mode 'minos'
     MINUIT2 errdef: 1
```

Model parameter

no.	name	unit	value	eparab	eminus	eplus	var/fix
0	b0	[−]	−9.49124	0.108256	−0.109529	0.106556	varbl
1	bsex	[−]	−0.321917	0.0571409	−0.0571794	0.0571278	varbl
2	bcity	[−]	−0.142548	0.0653043	−0.0660609	0.0646366	varbl
3	ba1	[−]	2.10991	0.270428	−0.27007	0.275135	varbl
4	ba2	[−]	1.07312	0.207564	−0.211356	0.201848	varbl
5	be1	[1/yr]	0.00642684	0.0045847	−0.00458582	0.00460188	varbl
6	be2	[1/yr^2]	−0.000720687	0.000227256	−0.000230395	0.00022298	varbl
7	errpd1	[1/Gy]	0.609022	0.820995	−0.731733	0.9081	varbl
8	dth	[Gy]	0.364909	0.0924305	−0.089404	0.155685	varbl
9	errpd2	[1/Gy]	5.14098	0.955362	−0.895951	1.0145	varbl
10	pa	[−]	−1.6349	0.353397	−0.371613	0.34316	varbl
11	cen_a	[yr]	55	−	−	−	fixed
12	px	[1/yr]	0	−	−	−	fixed
13	cen_e	[yr]	30	−	−	−	fixed
14	msex	[−]	0	−	−	−	fixed

Model parameter (final vs. initial)

no.	name	unit	final	initial	change	rel. ch. [%]
0	b0	[−]	−9.49124	−9.49119	−4.53212e−05	0.000477508
1	bsex	[−]	−0.321917	−0.321908	−9.02527e−06	0.00280368
2	bcity	[−]	−0.142548	−0.142564	1.55102e−05	−0.0108795
3	ba1	[−]	2.10991	2.10997	−5.84172e−05	−0.00276863
4	ba2	[−]	1.07312	1.07306	6.38583e−05	0.00595105
5	be1	[1/yr]	0.00642684	0.00642739	−5.5471e−07	−0.00863041
6	be2	[1/yr^2]	−0.000720687	−0.000720677	−1.04135e−08	0.00144496
7	errpd1	[1/Gy]	0.609022	0.60931	−0.000288369	−0.0473272
8	dth	[Gy]	0.364909	0.364875	3.44752e−05	0.00944849
9	errpd2	[1/Gy]	5.14098	5.14126	−0.000282251	−0.00548991
10	pa	[−]	−1.6349	−1.63495	4.78048e−05	−0.00292393
11	cen_a	[yr]	fixed	55		
12	px	[1/yr]	fixed	0		
13	cen_e	[yr]	fixed	30		
14	msex	[−]	fixed	0		

```
  Correlation matrix (of 11 variable parameters)
    0
    1   -0.053129807
    2     0.26947894    0.008844545
    3    -0.40306467   -0.078325787    0.031474253
    4    -0.20686757   -0.050219795   0.0050716855     0.36050601
    5     0.21499301   0.0090445639    0.073435081    -0.51289178    -0.17167312
    6    -0.51503516     0.13536433   -0.017249497    0.095401869    -0.15784814    -0.11864616
    7    -0.32379423   0.0036360068     0.10373654     0.21302329    -0.09213324  -0.0013154195   0.0055111397
    8    -0.10372576    0.021033529    0.034650101    0.078337267   -0.041028083    0.007613884   0.0028652683     0.57548046
    9    -0.42478742    0.051194884    0.012151625     0.13549553      0.1208261   0.0056966406    0.048707283     0.25884187
     0.43036441
   10   -0.037141981    0.024175694   -0.020556908    -0.36133543      0.5465256    0.020236492  0.00087861095    -0.13080706
   -0.058248783     0.20400457

-- O/E analysis
   Checking sums for persons, person years and cases
                         Stratification   O/E analysis
          Total persons:          86611          86611
     Total person years:     3.29428e+06    3.29428e+06
   Total observed cases:            318            318
   Total expected cases:        317.995        317.995
               Deviance:        2670.91        36.8212

   Cases
     Excess:     93.2
   Baseline:    224.8
      Total:    318.0

   Categories
        name     unit  id  size  used
     agexcat       yr   4     4     4
      agecat       yr   5     4     4
    marrow10       Gy   7     5     4

   Number of Poisson cells
                 Stratification   O/E analysis
   Total cells:           27720             64
    Used cells:           53782             40

   Poisson cells
   cell subjects         pyr  cases      obs haz    ubnd 4      mean 4    ubnd 5      mean 5    ubnd 7      mean 7
      0    13057   101123.29      1  9.88892e-06        20     4.46854        20     14.8023       0.5   0.0939403
      1     3975   324053.35     15  4.62887e-05        20      9.1773        40     30.1791       0.5   0.0975599
      2     8104    57092.47      3  5.25463e-05        40     25.0347        40     35.0642       0.5    0.107059
      3        0   315836.96     12  3.79943e-05        20     9.39005        60     49.8374       0.5   0.0979589
      4     3305   205804.81      6  2.91538e-05        40     30.2796        60     50.1692       0.5    0.107652
```

5	9067	68352.41	6	8.77804e-05	60	44.8118	60	54.8787	0.5	0.109273
6	0	109490.15	13	0.000118732	20	13.5791	infty	65.7873	0.5	0.10176
7	0	209170.81	37	0.000176889	40	30.8783	infty	72.0007	0.5	0.107023
8	1997	191482.30	28	0.000146228	60	48.5002	infty	72.2454	0.5	0.108687
9	2786	27718.45	2	7.21541e-05	infty	65.2003	infty	77.626	0.5	0.102219
10	1207	8215.25	8	0.000973799	20	4.88333	20	15.0014	1.5	0.978151
11	737	36059.42	8	0.000221856	20	11.1884	40	30.3333	1.5	0.97297
12	969	7463.32	1	0.000133989	40	24.5702	40	34.8287	1.5	0.977193
13	0	35265.68	8	0.000226849	20	11.4733	60	49.8035	1.5	0.97049
14	378	24001.33	7	0.00029165	40	29.6972	60	50.1327	1.5	0.984573
15	1027	7870.86	2	0.000254102	60	44.6862	60	54.8289	1.5	0.967113
16	0	15045.50	4	0.00026586	20	14.8263	infty	66.3623	1.5	0.967312
17	0	22505.23	8	0.000355473	40	30.2714	infty	71.4529	1.5	0.982348
18	185	19764.19	11	0.000556562	60	48.1545	infty	71.7338	1.5	0.952927
19	195	1922.04	0	0	infty	64.4784	infty	77.0941	1.5	0.912676
20	348	2263.04	6	0.0026513	20	4.80715	20	14.9324	infty	2.60704
21	175	9511.55	5	0.000525677	20	11.1083	40	30.3187	infty	2.52412
22	226	1763.85	2	0.00113389	40	24.4504	40	34.77	infty	2.43736
23	0	8996.04	2	0.00022232	20	11.4226	60	49.6696	infty	2.51478
24	76	5225.31	8	0.00153101	40	29.2529	60	49.9794	infty	2.43729
25	209	1641.38	2	0.00121849	60	44.6789	60	54.7877	infty	2.43315
26	0	3341.08	4	0.00119722	20	14.7015	infty	66.0517	infty	2.46649
27	0	4238.19	5	0.00117975	40	29.4826	infty	70.5257	infty	2.42636
28	37	3425.07	4	0.00116786	60	47.9354	infty	70.7562	infty	2.46001
29	42	369.32	1	0.00270768	infty	63.679	infty	75.8466	infty	2.51524

Observed/expected comparison

cell	pyr			cases			hazard	cell dev.
		obs	exp	bsl	obs	exp	bsl	
0	101123.29	1	3.9	2.0	9.88892e-06	3.8348e-05	2.01954e-05	3.04518
1	324053.35	15	9.2	7.5	4.62887e-05	2.84748e-05	2.3118e-05	3.03105
2	57092.47	3	2.3	1.9	5.25463e-05	3.97874e-05	3.30524e-05	0.211992
3	315836.96	12	15.7	14.3	3.79943e-05	4.96888e-05	4.52183e-05	0.946863
4	205804.81	6	14.1	12.7	2.91538e-05	6.87509e-05	6.17864e-05	6.00369
5	68352.41	6	5.8	5.3	8.77804e-05	8.4677e-05	7.68893e-05	0.00768093
6	109490.15	13	10.8	10.1	0.000118732	9.81952e-05	9.215e-05	0.440546
7	209170.81	37	31.1	29.4	0.000176889	0.000148891	0.000140345	1.03809
8	191482.30	28	28.6	26.9	0.000146228	0.000149555	0.000140652	0.014283
9	27718.45	2	2.9	2.7	7.21541e-05	0.000104156	9.90622e-05	0.30575
10	8215.25	8	6.0	0.2	0.000973799	0.000732	1.98065e-05	0.59391
11	36059.42	8	8.4	0.8	0.000221856	0.000232836	2.30613e-05	0.0189708
12	7463.32	1	1.9	0.2	0.000133989	0.000258895	3.19175e-05	0.547098
13	35265.68	8	7.8	1.6	0.000226849	0.000219921	4.51129e-05	0.00761788
14	24001.33	7	7.3	1.5	0.00029165	0.000305478	6.19855e-05	0.0152535
15	7870.86	2	2.7	0.6	0.000254102	0.000347604	7.96126e-05	0.218569
16	15045.50	4	4.6	1.3	0.00026586	0.000305382	8.95002e-05	0.0805075
17	22505.23	8	9.8	3.1	0.000355473	0.000437276	0.000137661	0.368147

18	19764.19	11	8.7	2.8	0.000556562	0.000438342	0.00014187	0.580097
19	1922.04	0	0.5	0.2	0	0.000278928	0.000102803	1.07222
20	2263.04	6	5.8	0.0	0.0026513	0.00257575	1.97177e−05	0.00496609
21	9511.55	5	7.5	0.2	0.000525677	0.00079325	2.51391e−05	0.975554
22	1763.85	2	1.4	0.1	0.00113389	0.000772495	3.22072e−05	0.260247
23	8996.04	2	6.2	0.4	0.00022232	0.000691879	4.9177e−05	3.90717
24	5225.31	8	4.4	0.3	0.00153101	0.000836348	6.13025e−05	2.41456
25	1641.38	2	1.6	0.1	0.00121849	0.000958613	7.93333e−05	0.106407
26	3341.08	4	3.1	0.3	0.00119722	0.000914322	0.000101114	0.266228
27	4238.19	5	4.5	0.6	0.00117975	0.00107003	0.000132663	0.046132
28	3425.07	4	3.9	0.5	0.00116786	0.00114743	0.000139313	0.00123876
29	369.32	1	0.3	0.0	0.00270768	0.000928926	0.000121918	0.82578
